# Two-stage estimators for spatial confounding with point-referenced data

**DOI:** 10.1093/biomtc/ujaf093

**Published:** 2025-07-24

**Authors:** Nate Wiecha, Jane A Hoppin, Brian J Reich

**Affiliations:** Department of Statistics, North Carolina State University, Raleigh, NC 27607, United States; Department of Biological Sciences, North Carolina State University, Raleigh, NC 27607, United States; Department of Statistics, North Carolina State University, Raleigh, NC 27607, United States

**Keywords:** bias reduction, double machine learning, Gaussian process, semiparametric regression

## Abstract

Public health data are often spatially dependent, but standard spatial regression methods can suffer from bias and invalid inference when the independent variable is associated with spatially correlated residuals. This could occur if, for example, there is an unmeasured environmental contaminant associated with the independent and outcome variables in a spatial regression analysis. Geoadditive structural equation modeling (gSEM), in which an estimated spatial trend is removed from both the explanatory and response variables before estimating the parameters of interest, has previously been proposed as a solution but there has been little investigation of gSEM’s properties with point-referenced data. We link gSEM to results on double machine learning and semiparametric regression based on two-stage procedures. We propose using these semiparametric estimators for spatial regression using Gaussian processes with Matèrn covariance to estimate the spatial trends and term this class of estimators double spatial regression (DSR). We derive regularity conditions for root-*n* asymptotic normality and consistency and closed-form variance estimation, and show that in simulations where standard spatial regression estimators are highly biased and have poor coverage, DSR can mitigate bias more effectively than competitors and obtain nominal coverage.

## 1 INTRODUCTION

Public health data are often observational and exhibit spatial dependence, such as in environmental contaminations that may spread over a geographic region (Cressie, 1993). Spatial regression methods can improve efficiency, allow proper uncertainty quantification, and enhance predictive accuracy (Cressie, 1993). However, association between explanatory variables and latent functions of space in the response model can cause bias in estimated regression coefficients, and invalid statistical inference due to poor uncertainty quantification (Reich et al., 2006). This has often been termed “spatial confounding,” and was first observed in Clayton et al. (1993), discussed further in Reich et al. (2006) and Hodges and Reich (2010), and studied in other papers such as Paciorek (2010). The analogous issue in spline models has been discussed in Rice (1986) and Wood (2017). Several recent papers (Dupont et al., 2023; Gilbert et al., 2021; Khan and Berrett, 2024) have discussed definitions, causes, and effects of spatial confounding in attempts to unify varying accounts.

Methods proposed to deal with spatial confounding aim to reduce bias in linear regression parameter estimates, such as in Marques et al. (2022), Guan et al. (2022), and Schnell and Papadogeorgou (2020). Methods similar to our proposal are geoadditive structural equation modeling (gSEM; Thaden and Kneib, 2018), Spatial+ (Dupont et al., 2022), and a shift estimand, which does not assume a linear treatment effect, studied in Gilbert et al. (2021). gSEM subtracts estimated latent functions of space from the treatment and response, and regresses those residuals onto each other. Spatial+ subtracts an estimated function of space from the treatment variable and regresses the response onto those residuals and a spatial term modeled by a thin-plate spline. The shift estimand implemented in Gilbert et al. (2021) subtracts an estimated function of space from the treatment (in order to estimate a conditional density function) and response but without assuming a linear and additive effect of the explanatory variable. Identifiability in gSEM, Spatial+, the shift estimand, and our method is typically due to independent, non-spatial variation in the treatment and response. In this situation, Gilbert et al. (2024) also showed that the standard generalized least-squares (GLS), spline, and Gaussian process estimates are consistent even in some cases where they are misspecified due to spatial confounding, but noted that this does not imply variance estimates are accurate, and these estimators may converge at a rate slower than $n^{-1/2}$.

gSEM was initially proposed for areal spatial data, and its analog for point-referenced spatial data, based on Appendix B of Thaden and Kneib (2018), has not been studied thoroughly. The subject of this paper is point-referenced spatial data, so we use “gSEM” to refer to its point-referenced implementation. Dupont et al. (2022) state that “it is not immediately clear why the method works”; Dupont et al. (2023) state that “the bias reduction will only be successful under the assumption that the initial regressions successfully remove all spatial confounders.” gSEM has also lacked a closed-form variance estimate.

Literature on semiparametric regression (Andrews, 1994; Chernozhukov et al., 2018; Robinson, 1988) proves that under spatial confounding and additional regularity conditions, a class of estimators, including some nearly identical to gSEM (Robinson, 1988), can achieve $n^{-1/2}$ consistency and asymptotic normality, with a consistent closed-form variance estimate, even when not removing spatial confounding fully in initial regression estimates of spatial trends. Broadly speaking, these estimators first estimate the latent functions of space using non-parametric regression, subtract these estimates from the observed variables, and use those residuals to estimate the regression parameter in a second stage; the preliminary estimates of the latent functions need converge to the true values only slowly. Due to a form of orthogonality between the estimator and the preliminary estimates of the latent functions, the asymptotic variance of the estimator is unaffected by using these preliminary estimates. By either using sample-splitting as in Chernozhukov et al. (2018) or requiring additional smoothness conditions as in Andrews (1994), asymptotic bias due to overfitting is controlled. A more detailed overview of the related semiparametric theory is presented in the Supplementary Materials Section S1.

Gilbert et al. (2021) showed that a double machine learning (DML) estimator can address spatial confounding using a purely non-parametric estimator of the average effect of a $\delta$-shift in a scalar treatment variable. We show that a semiparametric DML approach to spatial confounding, while restrictive in some ways, has substantial upsides. Gilbert et al. (2021) relies on theory originally meant for social network data (Ogburn et al., 2024), which with spatial data results in an unnecessarily restricted sample-splitting strategy whose validity is unproven. No closed-form variance estimate is available for the shift estimator. In our semiparametric approach, we are able to use fully random sample splits, which allows sharper, clearer regularity conditions due to better estimation of spatial trends. The parametric model for treatment effects allows closed-form variance estimation using standard empirical estimators, avoids having to specify a shift $\delta$, and easily incorporates multiple treatment variables. Our approach avoids estimating a density function, sometimes a computational difficulty. Finally, most methods for spatial confounding and spatial regression assume a linear model, so we continue to improve understanding of this common model.

We apply these semiparametric estimators to spatial confounding scenarios, using Gaussian Process (GP) regression to estimate the latent functions of space, a procedure also known as Kriging. We primarily rely on methods and theory from Chernozhukov et al. (2018), extending Chernozhukov et al.’s partially linear model to a vector treatment variable. As the two-stage estimators are termed DML in Chernozhukov et al. (2018), we refer to their narrower use to address spatial confounding as double spatial regression (DSR). In simulations of spatial confounding, we show that DSR can provide superior performance in severe confounding scenarios. Finally, we analyze the association between 5 per- and polyfluoroaklyl substances (PFAS) and thyroid stimulating hormone (TSH) levels in blood using DSR.

## 2 DOUBLE SPATIAL REGRESSION

### 2.1 Model and notation

For $i\in \lbrace 1,...,n\rbrace$, let $Y_i\in \mathbb {R}$ be the response variable, ${\bf A}_i = (a_{i1}, a_{i2}, ... , a_{i\ell })^T \in \mathbb {R}^{\ell }$ be the treatment variables, ${\bf Z}_i = (z_{i1}, z_{i2}, ..., z_{iv})^T \in \mathbb {R}^v$ be the covariates, and ${\bf S}_i$ be the spatial location contained in spatial domain $\mathcal {S}\subset \mathbb {R}^d$. The assumed model for $j = 1, ..., \ell$ is


(1) \begin{eqnarray*}
Y_i = {\bf A}_i^T\boldsymbol {\beta }_0 + {\bf Z}_i^T\boldsymbol{\theta }_{00} + g_0({\bf S}_i) + U_i,
\end{eqnarray*}

(2) \begin{eqnarray*}
A_{ij} = {\bf Z}_i^T\boldsymbol{\theta }_{0j} + m_{0j}({\bf S}_i) + V_{ij},
\end{eqnarray*}

where $\boldsymbol {\beta }_0$ is the regression coefficient vector of interest. The vectors $\boldsymbol{\theta }_{00}, ..., \boldsymbol{\theta }_{0\ell }$ and the vector of functions $\eta _0=(g_0, m_{01}, ..., m_{0\ell })$ are all nuisance parameters. $U_i$ and $V_{ij}$ are random error terms such that $E(U_i| {\bf A}_i, {\bf Z}_i, {\bf S}_i) = E(V_{ij}|{\bf Z}_i, {\bf S}_i) = 0$. Spatial confounding can be thought of as the presence of an unmeasured confounder with spatial structure, which is stochastically dependent with ${\bf A}_i$ and which affects the conditional expectation of $Y_i$ (Thaden and Kneib, 2018). Spatial confounding is present in (1) and (2) unless the functions $g_0({\bf S}_i)$ and $m_{0j}({\bf S}_i)$ are orthogonal with respect to the distribution of ${\bf S}_i$. If they are not orthogonal, standard spatial models will exhibit finite-sample bias and inaccurate variance estimation (Paciorek, 2010). For the theoretical results, we assume a random spatial design, but our empirical results verify that the proposed method can perform well if the ${\bf S}_i$ are defined deterministically. Assume ${\bf Y}$ and the columns of ${\bf A}$ are centered with mean 0.

### 2.2 DSR estimator

DSR estimates the spatial trends $g_0, m_{01}, ..., m_{0\ell }$ and covariate effects ${\bf Z}^T\boldsymbol{\theta }_{00}$, ${\bf Z}^T\boldsymbol{\theta }_{01}$,..., ${\bf Z}^T\boldsymbol{\theta }_{0\ell }$, subtracts those from ${\bf Y}, {\bf A}_{\cdot 1}, ..., {\bf A}_{\cdot \ell }$, and then uses these residuals to estimate $\boldsymbol {\beta }_0$. The estimates of $g_0, m_{01}, ..., m_{0\ell }$, and ${\bf Z}^T\boldsymbol{\theta }_{00}$, ${\bf Z}^T\boldsymbol{\theta }_{01}$,..., ${\bf Z}^T\boldsymbol{\theta }_{0\ell }$, are obtained by cross-fitting: for example, to estimate the values of $g_0$ for the data in fold *k*, an estimate $\widehat{g}_0$ of the function $g_0$ is obtained using the data not in fold *k*, and $\widehat{g}_0$ is then evaluated on the data in fold *k*.

For cross-fitting, the full data set is randomly (we use a completely random sample) partitioned into *K* folds each of size $\frac{n}{K}$, assumed to be an integer. Let $f_i \in \lbrace 1, ..., K\rbrace$ denote the fold assignment of observation *i*. For $k = 1, ..., K$, let ${\bf k}= \lbrace i: f_i = k\rbrace$ denote the set of indices assigned to fold *k* and ${\bf k}^C = \lbrace i: f_i \ne k\rbrace$ denote the set of indices assigned to the complement of fold *k*. Then, for example, ${\bf Y}_{{\bf k}}$ is the vector of elements of ${\bf Y}$ assigned to the *k*th fold and ${\bf Y}_{{\bf k}^C}$ is the vector of elements of ${\bf Y}$ not assigned to the *k*th fold. For a generic matrix ${\bf X}\in \mathbb {R}^{n \times p}$, the *i*th row of ${\bf X}$ is denoted by ${\bf X}_i$, the submatrix consisting of those rows of ${\bf X}$ whose indices are in ${\bf k}$ is denoted by ${\bf X}_{{\bf k}}$ (and likewise for ${\bf k}^C$), the *j*th column is denoted by ${\bf X}_{\cdot j}$, and the *j*th column vector of the submatrix corresponding to the *k*th fold is denoted by ${\bf X}_{{\bf k},j}$. Denote the full data as ${\bf W}$, the data in fold *k* as ${\bf W}_{{\bf k}}$, and the data in the complement of fold *k* as ${\bf W}_{{\bf k}^C}$.

Universal Kriging using the Matèrn correlation function is used to estimate $g_0, m_{01}, ..., m_{0\ell }$ (Stein, 1999). The Matèrn correlation function is


(3) \begin{eqnarray*}
C_{\gamma , \tau }({\bf S}_i, {\bf S}_j):&=& \frac{2^{1-\tau }}{\Gamma (\tau )}\biggl (\sqrt{2\tau }\frac{\Vert {\bf S}_i - {\bf S}_j\Vert _2}{\gamma }\biggr )^{\tau }\\
&& \times K_{\tau } \biggl (\sqrt{2\tau }\frac{\Vert {\bf S}_i - {\bf S}_j\Vert _2}{\gamma } \biggr ),
\end{eqnarray*}

where $K_{\tau }$ is the modified Bessel function of the second kind. The Matèrn correlation function has two parameters: $\gamma$ controls the range of spatial dependence and $\tau$ controls the smoothness (mean-square differentiability) of the process. A variance parameter $\omega ^2$ is typically defined so that $\omega ^2 C({\bf A}, {\bf B})$ is a covariance matrix.

For each fold *k* and for each of ${\bf Y}, {\bf A}_{\cdot 1}, ..., {\bf A}_{\cdot \ell }$, Models (1) and (2) are fit to ${\bf W}_{{\bf k}^C}$, using a spatial random effect with Matèrn covariance to model the spatial terms $g_0, m_{01}, ..., m_{0\ell }$, obtaining preliminary parameter estimates for each model fit. The preliminary estimates obtained on fold *k* for $\omega$, the correlation parameters used in (3), and the slope parameters used in (1) and (2) are denoted by a tilde and an additional subscript *k*; note that $\widetilde{\boldsymbol {\beta }_0}$, a preliminary estimate of $\boldsymbol {\beta }_0$, is included.

The universal Kriging equations used to estimate the spatial trends on fold *k* are


(4) \begin{eqnarray*}
\widehat{g}_0({\bf S}_{{\bf k}}) &=& \widetilde{\omega }_{0k}C_{\widetilde{\gamma }_{0k},\widetilde{\tau }_{0k}}({\bf S}_{{\bf k}}, {\bf S}_{{\bf k}^C})\\
&&\times \ \biggl(\widetilde{\omega }_{0k}C_{\widetilde{\gamma }_{0k}, \widetilde{\tau }_{0k}}({\bf S}_{{\bf k}^C}, {\bf S}_{{\bf k}^C}) + \widetilde{\sigma }_{0k}^2 {\bf I}\biggr )^{-1}\\
&& \times \ ({\bf Y}_{{\bf k}^C} - {\bf A}_{{\bf k}^C}^T\widetilde{\boldsymbol {\beta }}_{0k} - {\bf Z}_{{\bf k}^C}^T \widetilde{\boldsymbol{\theta }}_{00k})
\end{eqnarray*}

(5) \begin{eqnarray*}
\widehat{m}_{0j}({\bf S}_{{\bf k}}) &=& \widetilde{\omega }_{jk}C_{\widetilde{\gamma }_{jk},\widetilde{\tau }_{jk}}({\bf S}_{{\bf k}}, {\bf S}_{{\bf k}^C})\\
&&\times \ \biggl (\widetilde{\omega }_{jk}C_{\widetilde{\gamma }_{jk},\widetilde{\tau }_{jk}}({\bf S}_{{\bf k}^C}, {\bf S}_{{\bf k}^C}) + \widetilde{\sigma }_{jk}^2 {\bf I}\biggr )^{-1}\\
&& \times \ ({\bf A}_{{\bf k}^C, j} -{\bf Z}_{{\bf k}^C}^T \widetilde{\boldsymbol{\theta }}_{0jk}),
\end{eqnarray*}

for $j=1,...,p$. For this working prediction model, we also assume homoskedasticity of all error terms, with variance parameters $\sigma ^2_0, \sigma ^2_1, ..., \sigma ^2_\ell$. The estimated covariate effects for fold *k* are ${\bf Z}_{{\bf k}}^T\widetilde{\boldsymbol{\theta }}_{00k}, {\bf Z}_{{\bf k}}^T\widetilde{\boldsymbol{\theta }}_{01k}, ..., {\bf Z}_{{\bf k}}^T\widetilde{\boldsymbol{\theta }}_{0\ell k}$.

To obtain universal Kriging estimates, we used the GpGp R package (Guinness, 2018), which estimates all covariance and slope parameters using restricted maximum likelihood (REML), and scales well to large samples by using the Vecchia approximation (Vecchia, 1988). Estimates are combined across folds to obtain $\widehat{g}_0({\bf S})$ and $\widehat{m}_{01}({\bf S}), ..., \widehat{m}_{0 \ell }({\bf S})$, and $\widehat{{\bf Z}^T\boldsymbol{\theta }_{00}}, \widehat{{\bf Z}^T\boldsymbol{\theta }_{01}}, ..., \widehat{{\bf Z}^T\boldsymbol{\theta }_{0 \ell }}$. Denote the DSR estimator by $\widehat{\boldsymbol{\beta }}_{DSR}$. Letting $\widehat{{\bf V}}_{\cdot j} = {\bf A}_{\cdot j} -\widehat{{\bf Z}^T\boldsymbol{\theta }_{0j}}- \widehat{m}_{0j}({\bf S})$, and $\widehat{{\bf U}} = {\bf Y}- {\bf A}^T\widehat{\boldsymbol {\beta }}_{DSR} - \widehat{{\bf Z}^T\boldsymbol{\theta }_{00}} - \widehat{g}_0({\bf S})$


(6) \begin{eqnarray*}
\widehat{\boldsymbol {\beta }}_{DSR} &= (\widehat{{\bf V}}^T{\bf A})^{-1}\widehat{{\bf V}}^T({\bf Y}- \widehat{{\bf Z}^T {\boldsymbol{\theta }}_{00}} - \widehat{g}_0({\bf S}))
\end{eqnarray*}

(7) \begin{eqnarray*}
\widehat{Var}(\widehat{\boldsymbol {\beta }}_{DSR}) \!=\! (\widehat{{\bf V}}^T{\bf A})^{-1} \left(\sum _{i=1}^n \widehat{U}_i^2\widehat{{\bf V}}_i \widehat{{\bf V}}_i^T\right)\left((\widehat{{\bf V}}^T{\bf A})^{-1}\right)^T,\\
\end{eqnarray*}

which are the estimators provided in Chernozhukov et al. (2018). The DSR algorithm is given by Algorithm 1, and a version without cross-fitting, more closely corresponding to Andrews (1994), is provided in Supplementary Materials Section S4.

**Algorithm 1:**
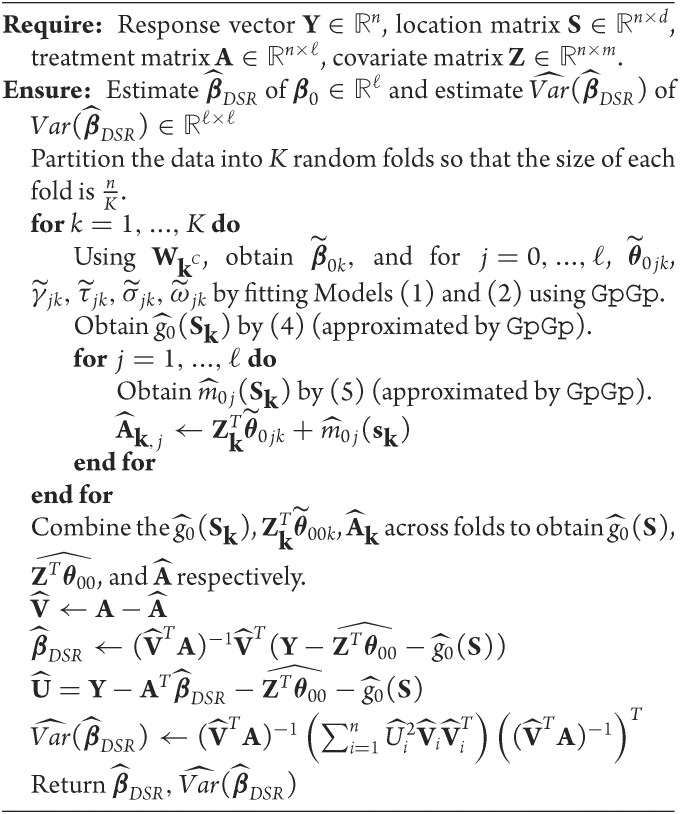
DSR estimation of β_0_ with cross-fitting

If cross-fitting is used, different random allocations of observations into folds will produce different estimates of $\boldsymbol {\beta }_0$. For a scalar $\beta _0$, Fuhr et al. (2024) recommend using enough folds that the estimates are relatively stable, and pick the median point and variance estimates out of as large a number of estimates as is feasible. The marginal median could be selected for each component of $\widehat{\boldsymbol {\beta }}_{DSR}$ if it has a length greater than 1.

In Supplementary Material Section S2, we prove root-*n* consistency and asymptotic normality for a different DSR estimator, also based on Chernozhukov et al. (2018), which more closely resembles gSEM in that in the first stage, ${\bf Y}$ is regressed directly onto ${\bf S}$, ignoring treatment variables. Covariates are also considered treatment variables, a limitation of our theory. We term this the “theoretical DSR” estimator and denote it by $\widehat{\boldsymbol {\beta }}_{TDSR}$. Instead of Matèrn covariance, $\widehat{\boldsymbol {\beta }}_{TDSR}$ estimates latent functions of space using Kriging with squared exponential covariance, with covariance parameter selection by a training-validation split (Eberts and Steinwart, 2013), which allows more thorough theoretical analysis. In summary, for $\mathcal {S} \subset \mathbb {R}^2$, the regularity conditions for $\widehat{\boldsymbol {\beta }}_{TDSR}$ require that the unknown functions of space are smoother than once-differentiable functions, and reside in $L_2(\mathbb {R}^2)\cap L_{\infty }(\mathbb {R}^2)$, as well as other conditions. Under Assumptions A1–A6 in Supplemental Materials Section S2, we show root-*n* consistency and asymptotic normality of $\widehat{\boldsymbol {\beta }}_{TDSR}$:

Theorem 1: If Assumptions A1–A6 are met, and $\widehat{\boldsymbol {\beta }}_{TDSR}$ and $\widehat{Var}(\widehat{\boldsymbol {\beta }}_{TDSR})$ are obtained by Algorithm S2, then

\begin{eqnarray*}
\sqrt{n}\boldsymbol{\Sigma }^{-1/2}(\widehat{\boldsymbol {\beta }}_{TDSR}- \boldsymbol {\beta }_{0}) \xrightarrow []{D} N(\mathbf {0}, \mathbf {I}_p), \\
\widehat{Var}(\widehat{\boldsymbol {\beta }}_{TDSR})^{-1/2}(\widehat{\boldsymbol {\beta }}_{TDSR} - \boldsymbol {\beta }_0) \xrightarrow []{D} N(\mathbf {0},\mathbf {I}_p),
\end{eqnarray*}

where $\boldsymbol{\Sigma }=E[{\bf V}_i{\bf V}_i^T]^{-1}E[U_i^2{\bf V}_i{\bf V}_i^T](E[{\bf V}_i{\bf V}_i^T]^{-1})$ is the approximate variance of $\widehat{\boldsymbol {\beta }}_{TDSR}$.

Theorem 1 does not apply directly to the estimator $\widehat{\boldsymbol {\beta }}_{DSR}$. Root-*n* consistency of $\widehat{\boldsymbol {\beta }}_{DSR}$ and asymptotic normality of $\widehat{Var}(\widehat{\boldsymbol {\beta }}_{DSR})^{-1/2}(\widehat{\boldsymbol {\beta }}_{DSR} - \boldsymbol {\beta }_0)$ require convergence of the estimates from (4) and (5) at rates of $o_P(n^{-1/4})$, along with correct model specification and further standard regularity conditions (Chernozhukov et al., 2018). Andrews (1994) provides sufficient conditions to forego cross-fitting, namely that both $(g_0, m_{01},...,m_{0\ell })$ and $(\widehat{g}_0, \widehat{m}_{01},...,\widehat{m}_{0\ell })$ obey additional smoothness conditions. These required results for GP regression with Matèrn correlation and hyperparameters estimated via REML are not established to our knowledge, so the use of $\widehat{\boldsymbol {\beta }}_{DSR}$ is theoretically justified, and cross-fitting can be skipped if desired, with assumptions about the convergence rates of $\widehat{g}_0, \widehat{m}_{01},...,\widehat{m}_{0\ell }$, and smoothness of both $(g_0, m_{01},...,m_{0\ell })$ and $(\widehat{g}_0, \widehat{m}_{01},...,\widehat{m}_{0\ell })$. Although equally thorough theoretical analysis is not possible, in simulations, $\widehat{\boldsymbol {\beta }}_{DSR}$ performed much better than $\widehat{\boldsymbol {\beta }}_{TDSR}$.

## 3 SIMULATION STUDY

### 3.1 Simulation study outline

In this simulation study, we evaluate several estimators’ performance in finite samples. We include the DSR estimator presented in Supplementary Materials Section 2.2 (Equations 6 and 7), which we refer to as “DSR” in this section, and the “theoretical DSR” estimator studied in Supplementary Materials Section S2 (Algorithm S2). Cross-fitting is used for all DSR results presented in this section; the Supplementary Materials Section S5 includes results for both versions of DSR without cross-fitting. DSR estimators with cross-fitting used $K=5$ folds. Different estimators of spatial trends, use of cross-fitting, and types of DSR estimators were used to isolate characteristics important to good empirical performance, and are detailed in the Supplemental Materials Section S4. Comparison methods were the geoadditive structural equation model (gSEM) of Thaden and Kneib (2018), implemented using splines for geostatistical data (Supplementary Materials Algorithm S1), Spatial+ of Dupont et al. (2022), the naive (unadjusted for spatial confounding) spatial linear mixed model (LMM), spline regression using generalized cross-validation (GCV) and REML to estimate the smoothing parameter for a smooth function of space (Wood, 2017), and ordinary least-squares (OLS). The non-parametric shift estimator studied in Gilbert et al. (2021) was tried but was not able to obtain estimates in most scenarios we used. Spatial+ removes a fitted spatial surface from the treatment variable, and then performs regression of the outcome onto the residuals with a thin-plate spline smooth over space. The naive spatial LMM is fitted using the R package GpGp (Guinness, 2018) and spline models fit using the R package mgcv (Wood, 2011). Simulations were carried out in R version 4.2.1 (R Core Team, 2023).

### 3.2 Data generating process

For our main simulations, we generate data from Gaussian processes in order to control the smoothness of the generated processes. Observations were drawn from multivariate normal distributions with selected covariances, equivalent to drawing a random function from a Gaussian process prior with that kernel. The data-generating process for the main simulations is similar to that used in Marques et al. (2022):


\begin{eqnarray*}
{\bf Y}&= \beta _0 {\bf A}+ {\bf Z}+ \boldsymbol{\epsilon }, \quad \epsilon _i \stackrel{i.i.d.}{\sim } N(0, \sigma ^2_Y), \\
{\begin{bmatrix}{\bf A}\\
{\bf Z}\end{bmatrix}} &\sim N {\begin{pmatrix}{\begin{bmatrix}\mathbf {0} \\
\mathbf {0} \end{bmatrix}}, {\begin{bmatrix}\boldsymbol{\Sigma }_{{\bf A}} +\sigma ^2_A{\bf I}& \rho \boldsymbol{\Sigma }_{{\bf A}}^{1/2}(\boldsymbol{\Sigma }_{{\bf Z}}^{1/2})^T \\
\rho \boldsymbol{\Sigma }_{{\bf Z}}^{1/2}(\boldsymbol{\Sigma }_{{\bf A}}^{1/2})^T & \boldsymbol{\Sigma }_{{\bf Z}} \end{bmatrix}} \end{pmatrix}},
\end{eqnarray*}

where ${\bf Y}, {\bf A}, {\bf Z}\in \mathbb {R}^n$, $\boldsymbol{\Sigma }_{{\bf A}}$ and $\boldsymbol{\Sigma }_{{\bf Z}}$ are spatial correlation matrices in $\mathbb {R}^{n \times n}$, and $\rho \in [-1, 1]$. ${\bf A}$ is the independent variable, $\beta _0$ is the parameter of interest, and ${\bf Z}$ is an unobserved spatial confounder. Since the main regularity conditions presented in Supplementary Materials Section S2 relate to the differentiability of the unknown functions of space, we used two spatial covariance matrices for both $\boldsymbol{\Sigma }_{{\bf A}}$ and $\boldsymbol{\Sigma }_{{\bf Z}}$ that generate realizations of smooth and rough, that is, more and less differentiable, functions:

- Smooth: Gneiting covariance function (Schlather et al., 2015) with range parameter $\gamma =0.2$, and variance 1.
- Rough: Matèrn covariance function with smoothness parameter $\tau =1.5$, range parameter $\gamma =0.072$, and variance 1.

The Gneiting covariance function approximates a squared exponential covariance function but is less prone to producing computationally singular covariance matrices (Schlather et al., 2015). These were both used as implemented in the R package geoR (Ribeiro et al., 2023). Range parameters were chosen so that the distance at which spatial correlation is equal to 0.05 were approximately equal. Example draws of spatial surfaces from these distributions are visualized in the Supplemental Materials Section S4.

For our main simulations, $n=1000$, $\rho =0.5$, $\sigma ^2_A=0.1^2$, $\sigma ^2_Y=1$, and $\beta _0=0.5$. The relative lack of i.i.d. variance in ${\bf A}$ provides less unconfounded variation in the treatment, causing severe spatial confounding bias in finite samples. Spatial locations were randomly sampled uniformly from $[0, 1]^2$. Several additional simulated scenarios generated data subject to heteroskedasticity, non-stationarity, non-Gaussianity of the confounder $Z_i$ or noise $\epsilon _i$, or considering deterministically defined latent functions of space $g_0$ and $m_0$ rather than random functions drawn from GP priors. These scenarios are discussed in the Supplementary Materials Section S4.

Four hundred Monte Carlo replications were performed for each main simulation scenario. Confidence intervals for Spatial+ and gSEM were obtained using 100 non-parametric bootstrap replicates. Methods were compared using their bias, standard error, mean squared error (MSE), coverage, and confidence interval length.

### 3.3 Simulation results

**FIGURE 1:**
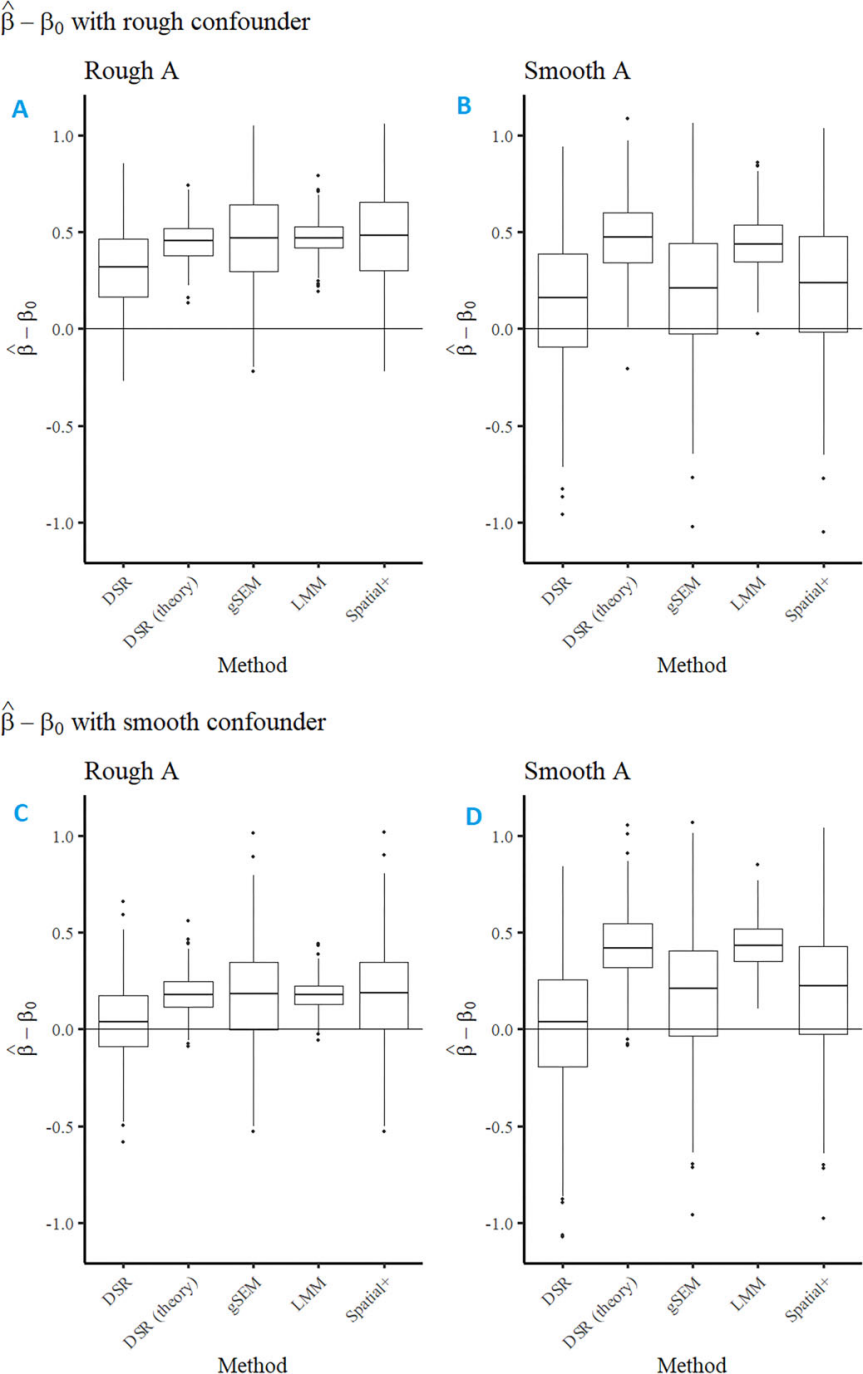
Sampling distribution of $\widehat{\beta } - \beta _0$ from DSR, theoretical DSR, gSEM, LMM, and Spatial+. In the top row (A, B), the unobserved confounder *Z* is rough, while in the bottom row (C, D), it is smooth. The treatment variable *A* is rough in the left column (A, C) and smooth in the right column (B, D). Linear mixed model, LMM; Double Spatial Regression, DSR; geoadditive structural equation modeling, gSEM.

Figure 1 displays sampling distributions of $\widehat{\beta }_0 - \beta _0$ for several scenarios and a subset of methods. Table 1 displays corresponding coverage and mean confidence interval lengths. DSR has the lowest bias and shortest confidence intervals of the methods with at least near-nominal coverage. Theoretical DSR performs fairly poorly in terms of bias, coverage, and confidence interval length. gSEM and Spatial+ are improvements over the linear mixed model, and achieve nominal coverage, but they have higher bias and wider confidence intervals than DSR. These scenarios were chosen to be particularly challenging cases of spatial confounding, in that there is little unconfounded variation in the treatment variable, so the linear mixed model performs poorly by all metrics.

**TABLE 1:** Coverage (mean confidence interval length) of 95% confidence intervals for DSR, theoretical DSR, gSEM, LMM, and Spatial+ in illustrative scenarios

*A*	*Z*	DSR	Theoretical DSR	gSEM	LMM	Spatial+
Rough	Rough	0.63 (0.79)	0.01 (0.43)	0.70 (1.25)	0.00 (0.33)	0.70 (1.26)
Smooth	Rough	0.93 (1.45)	0.37 (0.82)	0.95 (1.56)	0.09 (0.50)	0.95 (1.63)
Rough	Smooth	0.97 (0.78)	0.61 (0.41)	0.97 (1.24)	0.34 (0.30)	0.97 (1.24)
Smooth	Smooth	0.96 (1.46)	0.45 (0.79)	0.95 (1.55)	0.07 (0.46)	0.95 (1.62)

Scenarios varied by the smoothness of the treatment variable *A* and the unobserved spatial confounder *Z*. Linear mixed model, LMM; Double Spatial Regression, DSR; geoadditive structural equation modeling, gSEM.

Full simulation result tables are in the Supplementary Material Section S5, Tables S1–S13. In many scenarios, especially the deterministic scenarios where the latent functions of space were predefined, using cross-fitting proved beneficial. DSR implemented with spline regression without cross-fitting outperformed gSEM in many scenarios, indicating that direct estimation of $g_0({\bf S})$ rather than ignoring ${\bf A}$ in the first stage generally improves performance. Replacing the GP estimator in theoretical DSR by Kriging with Matèrn covariance and REML estimation greatly improved performance, but in a number of scenarios resulted in slight undercoverage, and sometimes worse bias than DSR. To study the robustness of DSR to the choice of correlation function, we applied DSR with Matèrn covariance smoothness fixed at 4.5. This resulted in slightly better estimates generally but this may be because many of our confounding scenarios were generated with smooth covariance functions.

In more difficult scenarios, such as those with rougher unobserved confounders, and the deterministic scenarios, DSR’s performance suffered but still outperformed other methods in bias, with slightly worse coverage than gSEM and Spatial+. The confounding scenario in which ${\bf A}$ and ${\bf Z}$ were generated with exponential covariance proved too difficult for any method to perform well and all suffered from substantial bias (Table S10 in the Supplementary Materials). When $\sigma ^2_A=1$, DSR had higher bias than gSEM and the shift estimator, although all methods had low bias in this scenario.

## 4 PFAS DATA ANALYSIS

PFAS are synthetic chemicals resistant to water and stains used in consumer products and manufacture of chemicals, many of which are frequently detected in humans due to resistance to degradation and solubility in water (National Academies of Sciences, Engineering, and Medicine, 2022). PFAS have been associated with thyroid disruption in humans, although results have been inconsistent (Coperchini et al., 2021; National Academies of Sciences, Engineering, and Medicine, 2022). Animal models indicate that PFAS can impact thyroid hormone function through a variety of mechanisms including thyroid hormone synthesis (Bali et al., 2024). Because PFAS reflect a broad class of chemicals, researchers are using in silico modeling to try to characterize these impacts (Baralić et al., 2024).

In Sun et al. (2016), high concentrations of a number of PFAS were detected in water at several collection points of the Cape Fear River in North Carolina (NC), the drinking water source for over a million people. One source of contamination is a fluorochemical manufacturing plant slightly downstream of Fayetteville, NC (Kotlarz et al., 2024; 2020). In 2017, the GenX Exposure Study began collecting data on NC residents in drinking water exposed communities in order to understand their patterns of exposure to PFAS and possible health effects. The data used in this analysis come from blood samples collected from study participants in the private well community near the chemical plant in October 2021. We study associations between five PFAS, because each was detected in at least 80% of our samples, and thyroid stimulating hormone (TSH).

The sample from this community is comprised of 98 adult women who obtain their water from private wells, who lived near the Fayetteville Works chemical plant, and who did not have thyroid disease at the time of data collection. Women with thyroid disease were excluded because thyroid medication can stabilize thyroid hormone levels. The outcome variable we studied was blood serum concentration of TSH. We focus on this outcome and community because the geographic area was relatively small (about 250 square miles), and the outcome and some exposures showed spatial dependence, so this analysis is a candidate for a spatial confounding adjustment. For example, other unmeasured pollutants might affect health outcomes and be correlated with the PFAS used in the analysis due to similar sources or spread. TSH was natural log-transformed to obtain approximately normal residuals.

Exposure variables were serum concentrations of five different PFAS detected in more than 80% of our sample: perfluoroheptanesulfonic acid (PFHpS), perfluorooctanoic acid (PFOA), perfluorononanoic acid (PFNA), perfluorooctane sulfonate (PFOS), and perfluorohexanesulfonic acid (PFHxS). The blood serum concentrations of PFAS and thyroid hormones were measured in the same samples. Wallis et al. (2023) summarize results from Li et al. (2018, 2022) and Zhang et al. (2013) that show fairly long half-lives of these chemicals, indicating that the measurements taken for these PFAS are good proxies of cumulative exposure. We controlled for several confounders that could be associated with both exposures and outcome: age, sex, race, smoking status and current alcohol consumption. Table 2 provides summary statistics of the continuous variables. The spatial proportion of residual variance is the variance of spatially-correlated residuals, divided by the total residual variance, in an LMM regressing that variable onto covariates. In sum, 39 members (40%) of the sample had ever smoked, 42 members (43%) currently drank, and 78 (80%) were White, 10 (10%) were Black, and 10 (10%) were other or multiple races. The median age was 59 years, with a mean of 56.9 and standard deviation of 14.7.

**TABLE 2:** Summary statistics of treatment and response variables used in the analysis of PFAS exposure’s association with TSH in 98 women from the private well community near the Fayetteville Works fluorochemical plant

Variable	Median	Mean	SD	Spatial prop. of resid. variance
PFHpS (ng/mL)	0.31	0.47	0.60	0.71
PFHxS (ng/mL)	1.96	2.34	1.58	0.00
PFNA (ng/mL)	0.55	0.68	0.54	0.33
PFOS (ng/mL)	5.87	7.02	5.16	0.17
PFOA (ng/mL)	2.17	2.50	1.79	0.46
TSH (uIU/mL)	1.34	1.51	0.71	0.41

The spatial proportion of residual variance is the variance of spatially correlated residuals, divided by the total residual variance, in a spatial linear mixed model regressing that variable onto covariates. TSH was log-transformed before estimating the spatial proportion of residual variance. PFAS, per- and polyfluoroalkyl substances; TSH, thyroid-simulating harmone.

DSR may offer an improved ability to analyze the effects of spatially-correlated chemical exposures, which may otherwise be difficult to estimate without adjustment for spatial confounding in standard models. DSR’s regularity conditions, while informative as to the general strength of the method, are not possible to check in practice, as they are assumptions on unknown functions of space. We recommend simply comparing model estimates from DSR and the LMM, and possibly OLS. A change to the statistical significance of results, or a change to point estimates practically relevant to the application, may indicate that spatial confounding was present in the LMM and adjusted for more successfully by DSR.

We estimated the joint associations between the five PFAS and TSH using non-spatial linear regression (OLS), a spatial LMM estimated with GpGp using Matèrn covariance, gSEM, Spatial+, and the DSR estimator with cross-fitting in Section 2.2. A large number of folds, i.e. 45, was used in the DSR estimator to improve stability of estimates across random splits; the marginal median estimates, and corresponding variance estimates, were chosen from 11 runs with different random sample splits. Linear regression diagnostics showed that an assumption of a linear association of all PFAS serum concentrations with log(TSH) was reasonable.

**FIGURE 2:**
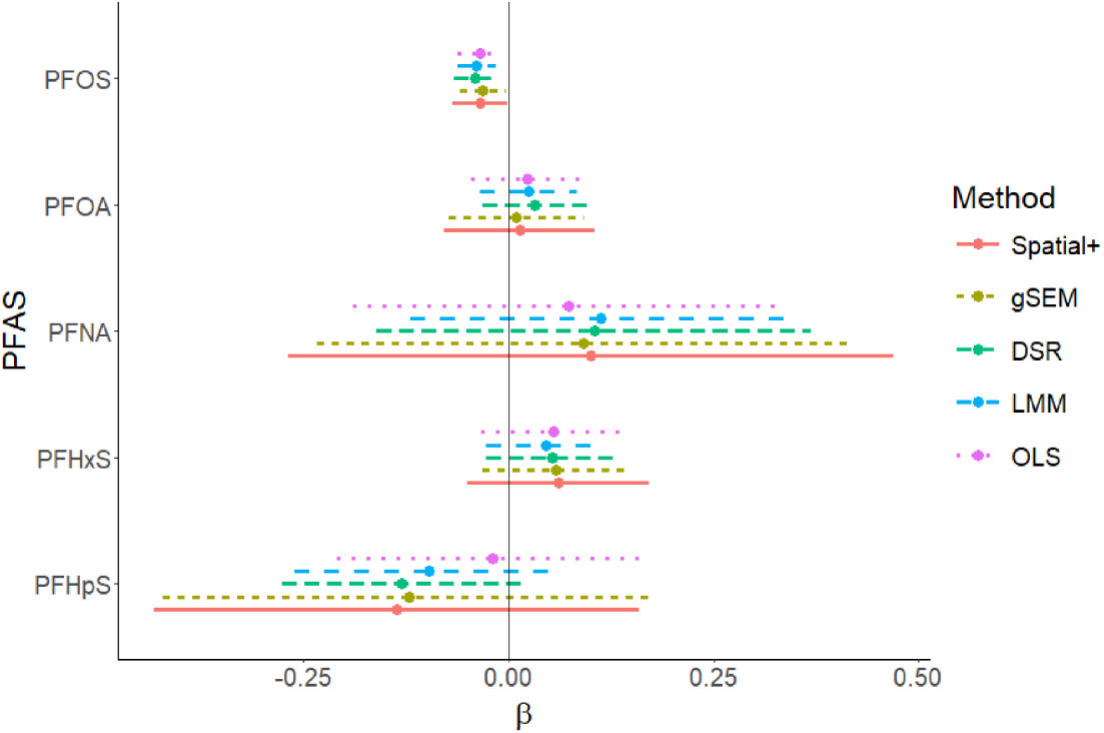
Forest plot of point estimates and 95% confidence intervals, based on the limiting normal distribution (OLS, LMM, DSR) or bootstrap estimation (Spatial+, gSEM), for regression coefficients $\beta$ for each PFAS. Results are presented for each method: OLS, LMM, DSR, gSEM, and Spatial+. Of the five PFAS, PFHpS had the strongest spatial autocorrelation, with approximately 70% of its residual variance explainable by a spatial random effect. DSR, double spatial regression; gSEM, geoadditive structural equation modeling; LMM, linear mixed model; OLS, ordinary least-squares; PFAS, per- and polyfluoroalkyl substances.

Regression analysis results, shown in Figure 2, indicate increasing magnitude and statistical significance of the $\beta$ parameter for PFHpS, from OLS to LMM to DSR. This trend of increasing magnitude and significance with increasing spatial confounding adjustment suggests the presence of spatial confounding with respect to PFHpS. About 70% of the residual variance in PFHpS was attributable to spatial variation, making estimation of its regression coefficient more susceptible to spatial confounding. gSEM and Spatial+ yielded similar point estimates to DSR, but with higher uncertainty, in line with simulation results with noisy exposure variables (Table S9).

## 5 DISCUSSION

DSR is a method to estimate linear regression coefficients using point-referenced data that may be spatially confounded. In simulated severe confounding scenarios, DSR outperformed competing methods in bias and coverage. We derived explicit regularity conditions that govern asymptotic performance of a slightly different DSR estimator. These regularity conditions are fairly unrestrictive but this “theoretical DSR” estimator performed poorly in simulations of severe confounding. DSR also allows closed-form variance estimation, which many competing methods have lacked. Based on simulation results, we recommend using the DSR estimator from Section 2.2 with cross-fitting.

We linked the problem of spatial confounding to existing results in the semiparametric regression literature, which has provided insight into why gSEM is able to reduce bias compared to the naive spatial linear mixed model, and studied DML estimators (which we termed DSR in our narrower application) that outperform others proposed so far. These existing results explain when and why these types of two-stage estimators are able to correct for bias, even when the initial non-parametric regression estimates converge to the true functions slowly. Simulation results indicate that both use of GP regression using Matèrn covariance, and direct estimation of the function $g_0$, as opposed to $h_0$, which marginalizes over the distribution of $A_i$, improved DSR’s estimates compared to gSEM in many scenarios.

DSR is similar in some ways to the method studied in Gilbert et al. (2021). This shift estimator was well suited to estimating the average treatment effect when treatment effects were heterogeneous. In many practical situations, there are multiple treatment variables, and it is reasonable to assume a linear and additive association between treatment(s) and response. In this situation, DSR is able to estimate multiple linear regression coefficients jointly, with their covariance matrix in closed-form. Gilbert et al. (2021) assumed increasing-domain asymptotics, while we assumed a fixed spatial domain.

Gilbert et al. (2021) also suggested using spatially-independent sample splits for cross-fitting, under an assumption of local spatial covariance. With this type of sample splitting, the conditional mean or density functions of the response and treatment variables, conditional on spatial location, are estimated using points in one subset of the spatial domain, and those functions’ realized values estimated on a different subset of the spatial domain such that the points in the prediction set are spatially independent from the points in the training set. It is not clear that these estimates can converge to their true values when the training and prediction sets are spatially independent, since the goal is to learn functions of space; deriving conditions on the covariance functions generating the spatially dependent data that are sufficient to achieve root-*n* asymptotic normality and consistency when using spatially independent splits could verify when this strategy can be used.

Further research could analyze convergence rates of functions estimated with Gaussian processes, when hyperparameters are selected using maximum likelihood or REML rather than training-validation splits; the only work we are aware of doing this, although with noiseless observations, is Karvonen et al. (2020). This would help yield more explicit regularity conditions for the main DSR estimator we proposed. Approaches to areal data should also be investigated. Finally, extensions to other types of semiparametric models, including weighted and non-linear regression, are discussed in Robinson (1988), Andrews (1994), and Chernozhukov et al. (2018).

## Supplementary Material

ujaf093_Supplemental_Files Web Appendices and Tables referenced in Sections 1, 2.2, 3.1, 3.2, and 3.3, and code for DSR estimators, simulation studies, and data analysis, are available with this paper at the Biometrics website on Oxford Academic. Code is also available on GitHub at https://github.com/nbwiecha/Double-Spatial-Regression.

## Data Availability

The data that support the findings in this paper cannot be shared publicly, for the privacy of individuals that participated in the study. The data will be shared on reasonable request to the corresponding author.
