# Supplementary material for: Two-stage estimators for spatial confounding with point-referenced data
Source: Biometrics. 2025 Jul 24;81(3):ujaf093. doi: 10.1093/biomtc/ujaf093 (PMC12288666; doi:10.1093/biomtc/ujaf093)
Supplement: ujaf093_Supplemental_Files — Web Appendices and Tables referenced in Sections 1, 2.2, 3.1, 3.2, and 3.3, and code for DSR estimators, simulation studies, and data analysis, are available with this paper at the Biometrics website on Oxford Academic. Code is also available on GitHub at https://github.com/nbwiecha/Double-Spatial-Regression. [file ujaf093_supplemental_files.zip › Two_stage_Estimators_Supp_Final.pdf]

Supplementary Materials for Two-Stage Estimators for Spatial Confounding with Point-Referenced Data

Nate Wiecha[1,*], Jane Hoppin[2], and Brian J. Reich[1]

[1]Department of Statistics, North Carolina State University, Raleigh, North Carolina, U.S.A

[2]Department of Biological Sciences, North Carolina State University, Raleigh, North Carolina, U.S.A

*nbwiecha@ncsu.edu

S1 Overview of related semiparametric theory

S1.1 Overview of related semiparametric theory

This section briefly summarizes the semiparametric literature on root-n consistency and asymptotic normality of estimators similar to gSEM. More complete explanations can be found in Andrews (1994) and Chernozhukov et al. (2018), from which this summary draws heavily. For $i \in \{1, ..., n\}$, let $Y_i \in \mathbb{R}$ be the response variable, A_i be the treatment variable, and $\mathbf{S}_i$ be the spatial location contained in spatial domain $\mathcal{S} \subset \mathbb{R}^d$. A simple model is:

$$\begin{aligned} Y_i &= A_i\beta_0 + g_0(\mathbf{S}_i) + U_i \\ A_i &= m_0(\mathbf{S}_i) + V_i. \end{aligned} \tag{1}$$

For illustration in this section we ignore covariates and assume A_i is a scalar. In (1), β_0 is the regression coefficient of interest, g_0 and m_0 are unknown functions treated as nuisance parameters, and U_i and V_i are error terms with finite, non-zero variance such that $E(U_i|A_i, \mathbf{S}_i) = 0$ and $E(V_i|\mathbf{S}_i) = 0$ for $i = 1, ..., n$. Denote by $h_0(\mathbf{s}) := E(Y_i|\mathbf{S}_i = \mathbf{s})$ (marginalizing over A_i), and let η_0 denote the vector of functions (g_0, m_0) or (h_0, m_0).

Model (1) can result in invalid inference due to spatial confounding if g_0, m_0 result in dependence between $\mathbf{A}$ and $g_0(\mathbf{S})$. For example, if $g_0 = m_0$, this corresponds to an unmeasured confounder which is a function of space affecting both $Y_1, ..., Y_n$ and $A_1, ..., A_n$, and in a spatial linear mixed model, this dependence is typically ignored. For example, spatial random effects are commonly used to implement a smoother to model the unknown function g_0, but are usually marginalized into the conditional variance of $\mathbf{Y}$ without consideration of dependence with $\mathbf{A}$ (Paciorek, 2010). Alternatively, some basis functions used to model g_0 may be collinear with $\mathbf{A}$, in which case penalized estimation of the coefficients on the basis functions leads to bias in estimation of β_0 (Reich et al., 2006).

Algorithm S1 Geoadditive Structural Equation Model (gSEM) estimation of β_0

Input: Response vector $\mathbf{Y} \in \mathbb{R}^n$, location matrix $\mathbf{S} \in \mathbb{R}^{n\times d}$, treatment vector $\mathbf{A} \in \mathbb{R}^n$
Output: Estimate $\widehat{\beta}_{gSEM}$ of $\beta_0 \in \mathbb{R}$

$\widehat{h}_0 \leftarrow$ Estimate of h_0 from a spatial regression model for $\mathbf{Y}$ onto $\mathbf{S}$, such as a spline regression
$\mathbf{R}_Y \leftarrow \mathbf{Y} - \widehat{h}_0(\mathbf{S})$
$\widehat{m}_0 \leftarrow$ Estimate of m_0 from a spatial regression model for $\mathbf{A}$ onto $\mathbf{S}$, such as a spline regression
$\mathbf{R}_A \leftarrow \mathbf{A} - \widehat{m}_0(\mathbf{S})$
$\widehat{\beta}_{gSEM} \leftarrow (\mathbf{R}_A^T\mathbf{R}_A)^{-1}\mathbf{R}_A^T\mathbf{R}_Y$, i.e., obtain $\widehat{\beta}_0$ by regressing the residuals $\mathbf{R}_Y$ onto the residuals $\mathbf{R}_A$
Return $\widehat{\beta}_{gSEM}$

The gSEM procedure is in Algorithm S1. Variance is typically estimated via bootstrap when gSEM is used in simulation studies (Guan et al., 2022; Gilbert et al., 2021). Aside from the method of estimating h_0 and m_0 and variance estimation, this is identical to the procedure considered in Robinson (1988). Intuitively, it is approximately orthogonalizing $\mathbf{Y}$ and $\mathbf{A}$ with respect to $\mathbf{S}$ and therefore removing effects of spatial confounding.

S1.1.1 Orthogonality of $\widehat{\beta}_{gSEM}$ and nuisance parameter estimates

To study $\widehat{\beta}_{gSEM}$'s asymptotic properties, note that $\widehat{\beta}_{gSEM}$ is equivalently defined as the solution to

$$\frac{1}{n}\sum_{i=1}^{n}\psi(Y_i, A_i, \mathbf{S}_i; \widehat{\beta}_{gSEM}, \widehat{\eta}_0) = 0$$

where $\psi(Y_i, A_i, \mathbf{S}_i; \beta, \eta) = \{Y_i - h(\mathbf{S}_i) - \beta(A_i - m(\mathbf{S}_i))\}(A_i - m(\mathbf{S}_i))$, $\eta = (h, m)$, $\widehat{\eta}_0 = (\widehat{h}_0, \widehat{m}_0)$, and $\widehat{h}_0$ and $\widehat{m}_0$ are preliminary estimates of the nuisance parameters h_0 and m_0. The score function ψ is used in Robinson (1988), and also studied in Andrews (1994) and Chernozhukov et al. (2018). This score function results in a form of orthogonality between $\widehat{\beta}_{gSEM}$ and $\widehat{\eta}_0$: under the model (1), replacement of η_0 by $\widehat{\eta}_0$ in $\frac{1}{n}\sum_{i=1}^{n} E[\psi(Y_i, A_i, \mathbf{S}_i; \beta_0, \eta_0)]$ has an effect that is $o_P(n^{-1/2})$ when $\widehat{\eta}_0$ is close to η_0 and $\widehat{h}_0$, $\widehat{m}_0$ each converge to their true values at $o_P(n^{-1/4})$ rate and obey smoothness conditions, and other regularity conditions are assumed (Andrews, 1994).[1] Alternatively, Chernozhukov et al. (2018) uses the term (near-) Neyman orthogonality. Neyman orthogonality means that the Gateaux functional derivative[2] with respect to η is 0 at the true nuisance parameter values:

$$\partial_\eta E[\psi(Y_i, A_i, \mathbf{S}_i; \beta_0, \eta_0)][\eta - \eta_0] = 0,$$

and in the case of near-Neyman orthogonality, that it is $o_P(n^{-1/2})$. Similarly, this indicates that close to η_0, there is little effect on $E[\psi(Y_i, A_i, \mathbf{S}_i; \beta_0, \eta_0)]$ when replacing η_0 by its estimate. The orthogonality property means that the estimates $\widehat{h}_0$ and $\widehat{m}_0$ can converge to h_0 and m_0 at rates slower than $o_p(n^{-1/2})$ and asymptotically this deviation from (h_0, m_0) does not affect affect the variance of $\widehat{\beta}_{gSEM}$ (Andrews, 1994).

S1.1.2 Stochastic equicontinuity or sample splitting

The orthogonality property of ψ must be paired with a means of ensuring that estimation error of η_0 does not cause asymptotic bias, primarily due to overfitting (Chernozhukov et al., 2018). This is achieved by a property of empirical processes called stochastic equicontinuity in Andrews (1994) and by sample

[1] Estimating the nuisance parameters using spline regression may not meet the sufficient conditions for good asymptotic behavior presented in (Andrews, 1994), which used nonparametric kernel regression estimators.

[2] Using the notation of Chernozhukov et al. (2018), the Gateaux derivative is defined as $D_r[\eta - \eta_0] := \partial_r\Big\{E[\psi(W;\beta_0, \eta_0 + r(\eta - \eta_0)]\Big\}$ for $r \in [0, 1)$, $\eta \in T$ where T is a convex subspace of a normed vector space, and $\partial_\eta E[\psi(W;\beta_0, \eta_0)] := D_0[\eta - \eta_0]$.

splitting in Chernozhukov et al. (2018). Stochastic equicontinuity follows from Donsker conditions (Belloni et al., 2017) which limit the complexity of η_0 and $\widehat{\eta}_0$. In Andrews (1994), these are primarily satisfied by placing a smoothness requirement on η_0 and $\widehat{\eta}_0$. In contrast, the sample-splitting approach of Chernozhukov et al. (2018) avoids requiring Donsker conditions, and therefore additional smoothness requirements. Since by using sample splitting Chernozhukov et al. (2018) allows estimation of η_0 by essentially any machine learning model, they term their method Double Machine Learning (DML). By using cross-fitting, the DML estimators of Chernozhukov et al. (2018) still use the full sample so do not lose power. We therefore rely on Chernozhukov et al. (2018) for our theoretical analysis of an estimator similar to gSEM, and term this estimator Double Spatial Regression since we are using Double Machine Learning with spatial regression.

S2 Double Spatial Regression estimator for theoretical analysis

In this section, we consider an algorithm similar to gSEM Appendix B (Thaden and Kneib, 2018) and the estimator in Robinson (1988), which allows derivation of explicit regularity conditions. For theoretical analysis, we combine treatment variables $\mathbf{A}_i$ and covariates $\mathbf{Z}_i$ into a combined vector $\mathbf{X}_i$ of regressors of length p. The model considered is then, for $i = 1, ..., n$ and $j = 1, ..., p$:

$$\begin{aligned} Y_i &= \mathbf{X}_i^T \boldsymbol{\beta}_0 + g_0(\mathbf{S}_i) + U_i, \qquad && E(U_i|\mathbf{X}_i, \mathbf{S}_i) = 0 \\ X_{ij} &= m_{0j}(\mathbf{S}_i) + V_{ij}, && E(V_{ij}|\mathbf{S}_i = 0). \end{aligned} \tag{2}$$

We treat covariates as treatment variables since existing results from machine learning theory, such as Eberts and Steinwart (2013), do not typically incorporate parametric adjustment for covariates into their analysis. We use these existing results to fully justify and analyze our estimator. We are not aware of available theoretical results that would allow parametric adjustment for covariates within our framework.

DSR uses Kriging (although other nonparametric estimators can be used) which requires a working correlation function be specified, and for theoretical analysis we use the squared exponential correlation function (Rasmussen and Williams, 2005):

$$C_\gamma(\mathbf{S}_i, \mathbf{S}_j) := \exp\left(-\frac{\|\mathbf{S}_i - \mathbf{S}_j\|_2^2}{\gamma^2}\right).$$

For matrix inputs $\mathbf{A}$ and $\mathbf{B}$ to a correlation function, $C(\mathbf{A}, \mathbf{B})$ denotes the correlation matrix where the element in row i and column j equals $C(\mathbf{A}_{i\cdot}, \mathbf{B}_{j\cdot})$.

Denote by $h_0(\mathbf{s})$ the conditional expectation $E(Y_i|\mathbf{S}_i = \mathbf{s})$ (note that this does not depend on $\mathbf{X}_i$). The number of elements in fold k is denoted by $|\mathbf{k}|$. On each fold k, Kriging (Stein, 1999) is used to obtain cross-fitted estimates for $h_0(\mathbf{S}_\mathbf{k})$ and $m_{0j}(\mathbf{S}_\mathbf{k})$ for $j = 1, ..., p$:

$$\widehat{h}_0(\mathbf{S}_\mathbf{k}) := C_{\gamma_{0k}}(\mathbf{S}_\mathbf{k}, \mathbf{S}_{\mathbf{k}^C})\Big(C_{\gamma_{0k}}(\mathbf{S}_{\mathbf{k}^C}, \mathbf{S}_{\mathbf{k}^C}) + |\mathbf{k}^C|\lambda_{0k}\mathbf{I}\Big)^{-1}\mathbf{Y}_{\mathbf{k}^C} \tag{3}$$

$$\widehat{m}_{0j}(\mathbf{S}_\mathbf{k}) := C_{\gamma_{jk}}(\mathbf{S}_\mathbf{k}, \mathbf{S}_{\mathbf{k}^C})\Big(C_{\gamma_{jk}}(\mathbf{S}_{\mathbf{k}^C}, \mathbf{S}_{\mathbf{k}^C}) + |\mathbf{k}^C|\lambda_{jk}\mathbf{I}\Big)^{-1}\mathbf{X}_{\mathbf{k}^C, j}. \tag{4}$$

Predictions are combined across folds to obtain $\widehat{h}_0(\mathbf{S})$ and $\widehat{m}_{01}(\mathbf{S}), ..., \widehat{m}_{0p}(\mathbf{S})$. The hyperparameters $\lambda_{0k}, ..., \lambda_{pk}$, $\gamma_{0k}, ..., \gamma_{pk}$ depend on k because our theoretical analysis requires that they are selected each time predictions are obtained (i.e., on each fold); they are selected using a training-validation split of $\mathbf{W}_{\mathbf{k}^C}$ (Eberts and Steinwart, 2013). The data in $\mathbf{W}_{\mathbf{k}^C}$ is split in two halves, a "training" half and a "validation" half. For each possible combination of hyperparameters (λ and γ) considered, the training half of $\mathbf{W}_{\mathbf{k}^C}$ is used to obtain predictions on the validation half of $\mathbf{W}_{\mathbf{k}^C}$. The pair of hyperparameters with lowest mean squared error (MSE) on the validation half of $\mathbf{W}_{\mathbf{k}^C}$ is selected for estimating $h_0, m_{01}, ..., m_{0p}$ evaluated at the locations corresponding to the data in $\mathbf{W}_\mathbf{k}$ (Eberts and Steinwart, 2013). This procedure is adopted in order to satisfy the requirements of Eberts and Steinwart (2013), which proves the convergence rate of certain GP regression estimates, in order to allow derivation of clear regularity conditions for DSR.

Letting $\widehat{\mathbf{V}}_{\cdot j} = \mathbf{X}_{\cdot j} - \widehat{m}_{0j}(\mathbf{S})$ and $\widehat{U}_i = Y_i - \widehat{h}_0(\mathbf{S}_i) - \widehat{\mathbf{V}}_i^T \widehat{\boldsymbol{\beta}}_0$, the DSR estimator and its approximate variance are:

$$\widehat{\boldsymbol{\beta}}_0 = (\widehat{\mathbf{V}}^T \widehat{\mathbf{V}})^{-1} \widehat{\mathbf{V}}^T (\mathbf{Y} - \widehat{h}_0(\mathbf{S})) \tag{5}$$

$$\widehat{Var}(\widehat{\boldsymbol{\beta}}_0) = (\widehat{\mathbf{V}}^T \widehat{\mathbf{V}})^{-1} \sum_{i=1}^{n} \left[\widehat{U}_i^2 \widehat{\mathbf{V}}_i \widehat{\mathbf{V}}_i^T \right] (\widehat{\mathbf{V}}^T \widehat{\mathbf{V}})^{-1}, \tag{6}$$

which are the estimators from Chernozhukov et al. (2018). The algorithm is presented in Algorithm S2. Note that this estimator is essentially the same as gSEM except that it uses sample splitting, the method of estimating the latent functions of space is not specified with gSEM, and gSEM has lacked a closed-form variance estimate; gSEM in turn is essentially identical to the estimator in Robinson (1988), except that Robinson (1988) uses nonparametric kernel regression estimators and provides a variance estimator.

Algorithm S2 Double Spatial Regression estimation of β_0 for theoretical study

Input: Centered response vector $\mathbf{Y} \in \mathbb{R}^n$, location matrix $\mathbf{S} \in \mathbb{R}^{n \times d}$, design matrix $\mathbf{X} \in \mathbb{R}^{n \times p}$ with centered columns.

Output: Estimate $\widehat{\boldsymbol{\beta}}_0$ of $\boldsymbol{\beta}_0 \in \mathbb{R}^p$ and estimate $\widehat{Var}(\widehat{\boldsymbol{\beta}}_0)$ of $Var(\widehat{\boldsymbol{\beta}}_0) \in \mathbb{R}^{p \times p}$

Randomly partition the data into K folds so that the size of each fold is $\frac{n}{K}$.

for $k = 1, ..., K$ **do**

Select the hyperparameters $\gamma_{0,k}, \lambda_{0,k} \in \mathbb{R}$ by a training-validation approach, using data in $\mathbf{k}^C$. The value of λ_{0k} is selected from an evenly-spaced grid of $\frac{1}{2n}$ values in $(0, 1]$, the lengthscale γ_{0k} is selected from an evenly-spaced grid of $\frac{1}{2n^{-1/4}}$ values in $(0, 1]$.

$$\widehat{h}_0(\mathbf{S}_{\mathbf{k}}) \leftarrow C_{\gamma_{0k}}(\mathbf{S}_{\mathbf{k}}, \mathbf{S}_{\mathbf{k}^C}) \left(C_{\gamma_{0k}}(\mathbf{S}_{\mathbf{k}^C}, \mathbf{S}_{\mathbf{k}^C}) + |\mathbf{k}^C| \lambda_{0k} \mathbf{I} \right)^{-1} \mathbf{Y}_{\mathbf{k}^C}$$

for $j = 1, ..., p$ **do**

Select the hyperparameters $\gamma_{j,k}, \lambda_{j,k} \in \mathbb{R}$ by a training-validation approach, using data in $\mathbf{k}^C$. The value of λ_{jk} is selected from an evenly-spaced grid of $\frac{1}{2n}$ values in $(0, 1]$, the lengthscale γ_{jk} is selected from an evenly-spaced grid of $\frac{1}{2n^{-1/4}}$ values in $(0, 1]$.

$$\widehat{m}_{0j}(\mathbf{S}_{\mathbf{k}}) \leftarrow C_{\gamma_{jk}}(\mathbf{S}_{\mathbf{k}}, \mathbf{S}_{\mathbf{k}^C}) \left(C_{\gamma_{jk}}(\mathbf{S}_{\mathbf{k}^C}, \mathbf{S}_{\mathbf{k}^C}) + |\mathbf{k}^C| \lambda_{jk} \mathbf{I} \right)^{-1} \mathbf{X}_{\mathbf{k}^C, j}$$

end for

end for

Combine the predictions from each fold to obtain $\widehat{h}_0(\mathbf{S}) \in \mathbb{R}^n, \widehat{\mathbf{m}}_0(\mathbf{S}) \in \mathbb{R}^{n \times p}$

$\widehat{\mathbf{V}} \leftarrow \mathbf{X} - \widehat{\mathbf{m}}_0(\mathbf{S})$

$\widehat{\mathbf{J}} \leftarrow (\widehat{\mathbf{V}}^T \widehat{\mathbf{V}})^{-1}$

$\widehat{\boldsymbol{\beta}}_0 \leftarrow \widehat{\mathbf{J}} \widehat{\mathbf{V}}^T (\mathbf{Y} - \widehat{h}_0(\mathbf{S}))$

$\widehat{Var}(\widehat{\boldsymbol{\beta}}_0) \leftarrow \widehat{\mathbf{J}} \left(\sum_{i=1}^n \left[-\widehat{\mathbf{V}}_i \widehat{\mathbf{V}}_i^T \widehat{\boldsymbol{\beta}}_0 + \widehat{\mathbf{V}}_i (Y_i - \widehat{h}_0(\mathbf{S}_i)) \right]^2 \right) \widehat{\mathbf{J}}^T$

Return $\widehat{\boldsymbol{\beta}}_0, \widehat{Var}(\widehat{\boldsymbol{\beta}}_0)$

S2.1 Double Spatial Regression regularity conditions

Our theoretical analysis uses the results on convergence rates of GP regression from Eberts and Steinwart (2013) and asymptotic properties of DML estimators from Chernozhukov et al. (2018), which require fast-enough convergence of estimates of the latent functions, to obtain explicit regularity conditions on the latent functions of space h_0 and $m_{01}, ..., m_{0p}$ under which DSR is root-n asymptotically normal and consistent. The observations $\mathbf{W}_i = (Y_i, \mathbf{X}_i, \mathbf{S}_i)$ are assumed to be i.i.d. from a probability distribution P with density function $p(w)$, and for a function f, $\|f\|_{P,q} := \{\int |f(w)|^q p(w) dw\}^{1/q}$. Note that although the $\mathbf{W}_i$ are assumed

to be drawn i.i.d. from P, the distribution of $Y_i|\mathbf{X}_i$ is still assumed to, in general, exhibit spatial dependence induced by marginalization over $\mathbf{S}_i$. The Euclidean norm is denoted $\|\cdot\|$, and $\|\cdot\|_p$ and $L_p(\mathbb{R}^d)$ are with respect to the Lebesgue measure.

As the assumptions govern the probability distribution P generating the i.i.d. random variables $\mathbf{W}_i$, regularity conditions are described for all draws, indexed by i, from P. The assumptions for the DSR estimator obtained by Algorithm S2 are:

A1) The data are generated by (2).

A2) The errors $U_i, \mathbf{V}_i$ are such that $E(U_i|\mathbf{X}_i, \mathbf{S}_i) = E(V_{ij}|\mathbf{S}_i) = 0$, for $j = 1, ..., p$, with $0 < E(U_i^2|\mathbf{S}_i) \leqslant C$ and $0 < E(V_{ij}^2|\mathbf{S}) \leqslant C$ for some constant $C > 0$ and all $\mathbf{S}_i \in \mathcal{S}$. Also, $E(U_i^2\mathbf{V}_i\mathbf{V}_i^T)$ and $E(\mathbf{V}_i\mathbf{V}_i^T)$ have minimum eigenvalues bounded away from 0. The errors U_i and V_{ij} are either contained in some interval or are normally distributed.

A3) The spatial locations $\mathbf{S}_i$ reside in a region $\mathcal{S}$ contained in a $\|\cdot\|$-unit ball in $\mathbb{R}^d$, and the boundary of $\mathcal{S}$ has P-probability 0. The marginal distribution P_S of $\mathbf{S}_i$ (derived from P) is absolutely continuous on $\mathcal{S}$ and has a density $p_S \in L_q(\mathbb{R}^d)$ for some $q \geqslant 1$.

A4) If U_i is bounded, then h_0 is such that $Y \in [-M_0, M_0]$ for some $M_0 > 0$, and if U_i is normally distributed then $h_0 \in [-1, 1]$. Similarly, if V_{ij} is bounded, then m_{0j} is such that $X_{ij} \in [-M_j, M_j]$ for some $M_j > 0$, and if V_{ij} is normally distributed then $m_{0j} \in [-1, 1]$, for $j = 1, ..., p$.

A5) The estimates $\widehat{h}_0, \widehat{m}_{01}, ..., \widehat{m}_{0p}$ are obtained using GP regression as described in Eberts and Steinwart (2013). The function $\widehat{h}_0$ is clipped so that if U_i is bounded, $|\widehat{h}_0| \leqslant M_0$ and if U_i is normally distributed, $|\widehat{h}_0| \leqslant \min\{1, 4\sqrt{C_0}\sqrt{\ln(n)}\}$ for some $C_0 > 0$ that exceeds $Var(U_i|\mathbf{S}_i)\,\forall \mathbf{S}_i \in \mathcal{S}$. Similarly, for $j = 1, ..., p$, $\widehat{m}_{0j}$ is clipped so that if V_{ij} is bounded, $|\widehat{m}_{0j}| \leqslant M_j$ and if V_{ij} is normally distributed, $|\widehat{m}_{0j}| \leqslant \min\{1, 4\sqrt{C_j}\sqrt{\ln(n)}\}$ for some $C_j > 0$ that exceeds $Var(V_{ij}|\mathbf{S})\,\forall \mathbf{S}_i \in \mathcal{S}$.

A6) For $j = 1, ..., p$, the functions m_{0j} reside in the Besov space $B^{\alpha_X}_{2s,\infty}$ where the smoothness order $\alpha_X > \frac{d}{2}$, $\alpha_X \geqslant 1$, and $\frac{1}{s} + \frac{1}{q} = 1$ and $s \geqslant 1$, and h_0 resides in the Besov space $B^{\alpha_Y}_{2s,\infty}$ where the smoothness order $\alpha_Y > \frac{d^2}{4\alpha_X}$ and $\alpha_Y \geqslant 1$. Furthermore, $h_0, m_{01}, ..., m_{p0} \in L_2(\mathbb{R}^d) \cap L_\infty(\mathbb{R}^d)$.

In the common scenario $d = 2$, a stronger but more interpretable alternative to Assumption A6 is that $h_0, m_{01}, ..., m_{0p}$ are each in $L_2(\mathbb{R}^d) \cap L_\infty(\mathbb{R}^d)$, each has at least two (weak) derivatives, and these functions and derivatives are all in $L_{2s}(\mathbb{R}^d)$. Per the discussion following Theorem 3.6 in Eberts and Steinwart (2013), $U_i, V_{i1}, ..., V_{ip}$ can follow other light-tailed distributions aside from normal.

Theorem 1 states that the DSR estimator in (5) is root-n asymptotically normal and consistent under the above assumptions.

Theorem 1 *If Assumptions A1 – A6 are met, and $\widehat{\boldsymbol{\beta}}_0$ and $\widehat{Var}(\widehat{\boldsymbol{\beta}}_0)$ are obtained by Algorithm S2, then*

$$\sqrt{n}\boldsymbol{\Sigma}^{-1/2}(\widehat{\boldsymbol{\beta}}_0 - \boldsymbol{\beta}_0) \xrightarrow{D} N(\mathbf{0}, \mathbf{I}_p), \text{ and}$$
$$\widehat{Var}(\widehat{\boldsymbol{\beta}}_0)^{-1/2}(\widehat{\boldsymbol{\beta}}_0 - \boldsymbol{\beta}_0) \xrightarrow{D} N(\mathbf{0}, \mathbf{I}_p),$$

where $\boldsymbol{\Sigma} = E[\mathbf{V}_i\mathbf{V}_i^T]^{-1}E[U_i^2\mathbf{V}_i\mathbf{V}_i^T](E[\mathbf{V}_i\mathbf{V}_i^T]^{-1})$ is the approximate variance of $\widehat{\boldsymbol{\beta}}_0$.

The proof of Theorem 1 is in the Supplementary Materials Section S6.

S2.2 Smoothness conditions

To provide intuition on Assumption A6, the following explanation of Besov spaces is paraphrased from Eberts and Steinwart (2013). Denote the ζ-th weak derivative $\partial^{(\zeta)}$ for a multi-index $\zeta = (\zeta_1, \zeta_2, ..., \zeta_d) \in \mathbb{N}^d$ with $|\zeta| = \sum_{i=1}^d \zeta_i$. With regard to a measure ν, the Sobolev space $W_p^\alpha(\nu)$ is defined as:

$$W_p^\alpha(\nu) := \{f \in L_p(\nu) : \partial^{(\zeta)} f \in L_p(\nu) \text{ exists for all } \zeta \in \mathbb{N}^d \text{ with } |\zeta| < \alpha\}.$$

Loosely speaking, $W_p^\alpha(\nu)$ is the space of functions with α weak derivatives, which all must have finite $L_p(\nu)$ norm. We refer to Eberts and Steinwart (2013) for a full definition of Besov spaces $B_{p,q}^\alpha$, but Besov spaces provide a finer scale of smoothness than the integer-ordered Sobolev spaces, and Sobolev spaces are contained in the Besov spaces:

$$W_p^\alpha(\mathbb{R}^d) \subset B_{p,q}^\alpha(\mathbb{R}^d)$$

for $\alpha \in \mathbb{N}, p \in (1,\infty), \max\{p,2\} \leqslant q \leqslant \infty$.

A stronger, but more interpretable alternative to the assumption that $h_0 \in B_{2s,\infty}^{\alpha_Y}$ and $m_{01},...,m_{0p} \in B_{2s,\infty}^{\alpha_X}$ in assumption A6 is that the m_{0j} reside in the integer-order Sobolev space $W_{2s}^{\alpha'_X}$ and h_0 resides in the integer-order Sobolev space $W_{2s}^{\alpha'_Y}$, where $\alpha'_X = \lceil \alpha_X \rceil$ and $\alpha'_Y = \lceil \alpha_Y \rceil$ and $\lceil a \rceil$ indicates the lowest integer greater than a. In the common scenario $d = 2$, the requirements on α_X and α_Y reduce to $\alpha_X > 1$ and $\alpha_Y > 1$, which for integer-ordered Sobolev spaces, loosely means that both h_0 and m_0 have at least two partial derivatives, or are smoother than functions with only one partial derivative.

S3 Additional algorithm

Below is the algorithm for DSR estimation without cross-fitting. As in the main paper, the assumed model for $j = 1,...,\ell$ is:

$$Y_i = \mathbf{A}_i^T \boldsymbol{\beta}_0 + \mathbf{Z}_i^T \boldsymbol{\theta}_{00} + g_0(\mathbf{S}_i) + U_i \tag{7}$$

$$A_{ij} = \mathbf{Z}_i^T \boldsymbol{\theta}_{0j} + m_{0j}(\mathbf{S}_i) + V_{ij}, \tag{8}$$

If cross-fitting is not used, the Universal Kriging equations are:

$$\widehat{g}_0(\mathbf{S}) = \widehat{\omega}_0^2 C_{\widehat{\gamma}_0,\widehat{\tau}_0}(\mathbf{S},\mathbf{S}) \left(\widehat{\omega}_0^2 C_{\widehat{\gamma}_0,\widehat{\tau}_0}(\mathbf{S},\mathbf{S}) + \widehat{\sigma}_0^2 \mathbf{I} \right)^{-1} (\mathbf{Y} - \mathbf{A}^T \widetilde{\boldsymbol{\beta}}_0 - \mathbf{Z}^T \widehat{\boldsymbol{\theta}}_0) \tag{9}$$

$$\widehat{m}_{0j}(\mathbf{S}) = \widehat{\omega}_j^2 C_{\widehat{\gamma}_j,\widehat{\tau}_j}(\mathbf{S},\mathbf{S}) \left(\widehat{\omega}_j^2 C_{\widehat{\gamma}_j,\widehat{\tau}_j}(\mathbf{S},\mathbf{S}) + \widehat{\sigma}_j^2 \mathbf{I} \right)^{-1} (\mathbf{A}_j - \mathbf{Z}^T \widehat{\boldsymbol{\theta}}_j), \tag{10}$$

where notation is as in the main paper, except that the subscript k indicating folds has been dropped as all parameter selections and predictions are obtained using the full dataset.

Algorithm S3 DSR estimation of $\boldsymbol{\beta}_0$ without cross-fitting

Input: Response vector $\mathbf{Y} \in \mathbb{R}^n$, location matrix $\mathbf{S} \in \mathbb{R}^{n \times 2}$, treatment matrix $\mathbf{A} \in \mathbb{R}^{n \times \ell}$, covariate matrix $\mathbf{Z} \in \mathbb{R}^{n \times m}$.
Output: Estimate $\widehat{\boldsymbol{\beta}}_{DSR}$ of $\boldsymbol{\beta}_0 \in \mathbb{R}^\ell$ and estimate $\widehat{Var}(\widehat{\boldsymbol{\beta}}_{DSR})$ of $Var(\widehat{\boldsymbol{\beta}}_{DSR}) \in \mathbb{R}^{\ell \times \ell}$
  Using $\mathbf{W}$, obtain $\widetilde{\boldsymbol{\beta}}_0$, and for $j = 0,...,\ell$, $\widetilde{\boldsymbol{\theta}}_{0j}$, $\widetilde{\gamma}_j, \widetilde{\tau}_j, \widetilde{\sigma}_j, \widetilde{\omega}_j$ by fitting Models (7) and (8) using `GpGp`.
  Obtain $\widehat{g}_0(\mathbf{S})$ by (9) (approximated by `GpGp`).
  for $j = 1,...,\ell$ **do**
    Obtain $\widehat{m}_{0j}(\mathbf{S})$ by (10) (approximated by `GpGp`).
    $\widehat{\mathbf{A}}_{\cdot,j} \leftarrow \mathbf{Z}^T \widetilde{\boldsymbol{\theta}}_{0j} + \widehat{m}_{0j}(\mathbf{S})$
  end for
  $\widehat{\mathbf{V}} \leftarrow \mathbf{A} - \widehat{\mathbf{A}}$
  $\widehat{\boldsymbol{\beta}}_{DSR} \leftarrow (\widehat{\mathbf{V}}^T \mathbf{A})^{-1} \widehat{\mathbf{V}}^T (\mathbf{Y} - \mathbf{Z}^T \widetilde{\boldsymbol{\theta}}_{00} - \widehat{g}_0(\mathbf{S}))$
  $\widehat{\mathbf{U}} = \mathbf{Y} - \mathbf{A}^T \widehat{\boldsymbol{\beta}}_{DSR} - \mathbf{Z}^T \widetilde{\boldsymbol{\theta}}_{00} - \widehat{g}_0(\mathbf{S})$
  $\widehat{Var}(\widehat{\boldsymbol{\beta}}_{DSR}) \leftarrow (\widehat{\mathbf{V}}^T \mathbf{A})^{-1} \left(\sum_{i=1}^n \widehat{U}_i^2 \widehat{\mathbf{V}}_i \widehat{\mathbf{V}}_i^T \right) \left((\widehat{\mathbf{V}}^T \mathbf{A})^{-1} \right)^T$
  Return $\widehat{\boldsymbol{\beta}}_{DSR}, \widehat{Var}(\widehat{\boldsymbol{\beta}}_{DSR})$

S4 Additional simulation details

Additional scenarios:

1. Cubed confounder: $Y_i \sim N(\beta A_i + Z_i^3, \sigma_Y^2)$.

2. Gamma errors in $\mathbf{Y}$: $Y_i = \beta A_i + Z_i + \phi_i, \phi_i = q[\Phi(\epsilon_i/\sqrt{3})]$, where q is the quantile function for the Gamma$(1, 1/\sqrt{3})$ distribution and Φ is the standard normal CDF, and $\epsilon_i \sim N(0, \sigma_Y^2)$.

3. "East-west" heteroskedasticity: $Y_i = \beta A_i + Z_i + S_{1i}\epsilon_i$, where S_{1i} is the first coordinate of $\mathbf{S}_i$ and $\epsilon_i \sim N(0, \sigma_Y^2)$.

4. "Middle-out" heteroskedasticity: $Y_i = \beta A_i + \sqrt{\frac{\omega(S_{1i})}{3}} Z_i + \sqrt{1 - \omega(S_{1i})}\epsilon_i$, where $\omega(S_{1i}) = \Phi(\frac{S_{1i}-0.5}{0.1})$ and $\epsilon_i \sim N(0, \sigma_Y^2)$.

The last three are borrowed from Huiying Mao and Reich (2023). Five further scenarios were considered.

1. Higher variance in $\mathbf{A}$: $\sigma_A^2 = 1$, causing less confounding bias.

2. Very rough processes: $\mathbf{\Sigma}_A$ and $\mathbf{\Sigma}_Z$ were Matèrn correlation matrices with smoothness 0.5, equivalent to exponential covariance, making adjustment more challenging due to very rough sample paths. The range parameter for the covariance function was 0.114 to have similar practical spatial range as the other scenarios.

3. Gridded spatial locations: Theory requires random spatial locations, but this illustrates the method with (very regular) fixed spatial locations.

4. Deterministic function of space, same for $\mathbf{A}$ and $\mathbf{Z}$: To avoid over-stating the effectiveness of DSR when the latent functions of space are generated and estimated using GPs, the data were generated using: $Y_i = \beta_0 A_i + g_0(\mathbf{s}_i) + \epsilon_{0i}$, and $A_i = m_0(\mathbf{s}_i) + \epsilon_{1i}$, where $g_0(\mathbf{s}_i) = m_0(\mathbf{s}_i) = \cos(10s_{i1})\sin(10s_{i2})$, $\epsilon_{0i} \overset{i.i.d.}{\sim} N(0, 1^2)$, and $\epsilon_{1i} \overset{i.i.d.}{\sim} N(0, 0.1^2)$.

5. Deterministic function of space, different for $\mathbf{A}$ and $\mathbf{Z}$: Similar to the previous scenario, but now $g_0(\mathbf{s}_i) = m_0(\mathbf{s}_i) + \sin(10s_{i1})\sin(10s_{i2})$ and m_0 is defined as in the previous scenario.

Plots of smooth, rough, and very rough simulated spatial surfaces are below in Figures 1, 2, and 3. These are examples of surfaces drawn from the distributions used to generate observations of the treatment variable $\mathbf{A}$ and the unobserved confounder $\mathbf{Z}$ in the simulation study. Since these surfaces are drawn randomly in each iteration of the simulation, these are only representative of the level of smoothness in each type of distribution used, and are not actual simulated datasets used. The data in the following plots are placed on a regular grid for easier visualization of the smoothnesses, rather than having spatial locations drawn randomly as in most of the simulated scenarios.

In implementation of gSEM and Spatial+, no adjustment was made in the bootstrapping procedure for spatial correlation between observations, or the tendency of GAMs to under-smooth in bootstrap samples described in Wood (2017). Spline models, gSEM, and Spatial+ used 300 spline basis functions.

S5 Full simulation results

In the following tables, the following methods were compared:

- OLS: ordinary least squares regression.
- LMM: Spatial linear mixed model, estimated using `GpGp` (Guinness, 2018).
- Spline (GCV): spline model estimated using `mgcv` (Wood, 2011), with smoothing parameter selected by generalized cross-validation, to minimize out-of-sample prediction error.

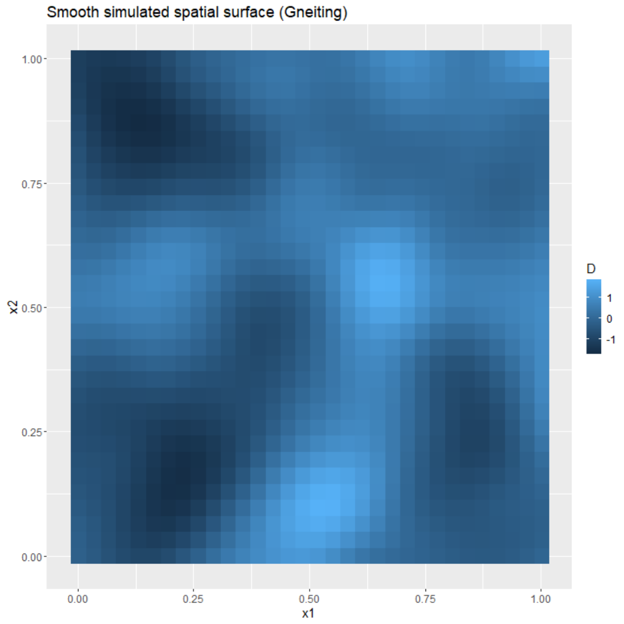

Figure 1: Smooth simulated spatial surface, drawn a multivariate normal distribution with Gneiting covariance, and range parameter 0.2, as used in the simulation study.

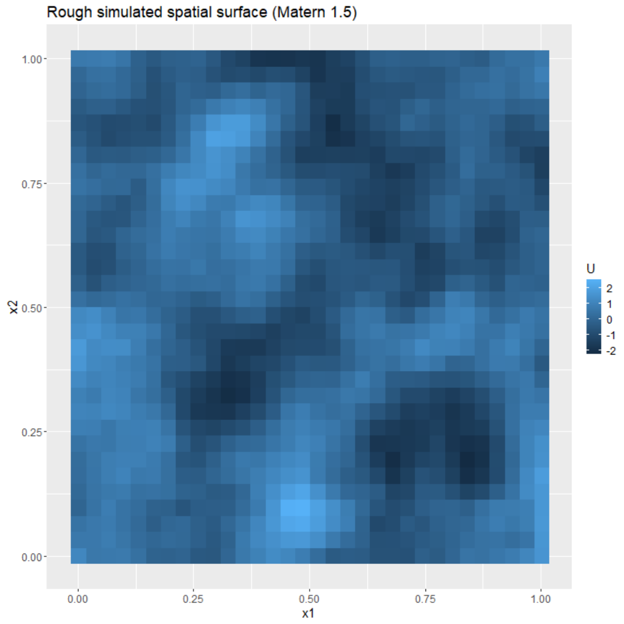

Figure 2: Rough simulated spatial surface, drawn a multivariate normal distribution with Matèrn covariance, smoothness parameter 1.5, and range parameter 0.072, as used in the simulation study.

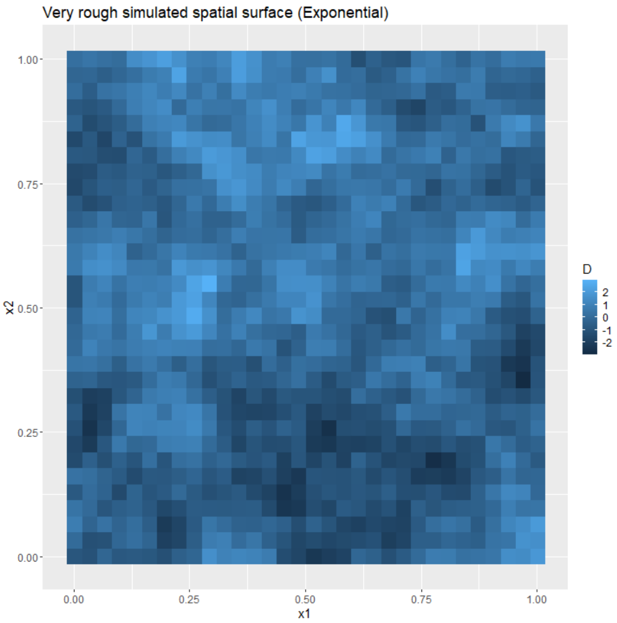

Figure 3: Very rough simulated spatial surface, drawn from a multivariate normal distribution with exponential covariance, and range parameter 0.114, as used in the simulation study.

- Spline (REML): spline model estimated using `mgcv`, smoothing parameter selected by restricted maximum likelihood (REML); Wood (2017) states that this might reduce bias when the parametric component is collinear with smooth term.
- Spatial+: the method of Dupont et al. (2022).
- gSEM: the point-referenced version of Thaden and Kneib (2018), described in Algorithm S1.
- Shift (BART): the shift estimand implemented in Gilbert et al. (2021), using Bayesian Additive Regression Trees (Chipman et al., 2010), or BART, to obtain the preliminary nonparametric estimates. BART estimates were obtained from the R package `dbarts` Dorie (2024). Results only presented in Table S9 due to inability to obtain estimates in other scenarios. Variance estimates not obtained due to relatively high expense of bootstrapping.
- DSR (theory): the theoretical DSR estimator described in Algorithm S2.
- DSR (theory, no crossfit): the theoretical DSR estimator described in Algorithm S2 but without crossfitting.
- DSR: The DSR estimator described in Algorithm 1.
- DSR: (no crossfit): the DSR estimator without crossfitting described in Algorithm S3.
- DSR (spline, no crossfit): the DSR estimator without crossfitting described in Algorithm S3 but with splines used instead of GP regression.
- DSR (theory, spline, no crossfit): the theoretical DSR estimator described in Algorithm S2 but without crossfitting, and with splines used instead of GP regression. This is equivalent to the implementation of gSEM used, except that it has a closed-form variance estimate.
- DSR (theory, GpGp): the theoretical DSR estimator described in Algorithm S2 but with GP regression implemented by the `GpGp` package (Guinness, 2018).

- DSR (Smooth covariance): The DSR estimator with crossfitting described in Algorithm 1, but with the smoothness term of the Matèrn covariance fixed at 4.5 for a smoother fitted function of space.
- DSR (Theory, smooth covariance): the theoretical DSR estimator described in Algorithm S2 but with GP regression implemented by the `GpGp` package (Guinness, 2018), and with the smoothness term of the Matèrn covariance fixed at 4.5 for a smoother fitted function of space.

Note that for theoretical DSR, we use a grid of possible γ values of size $\frac{1}{2n^{-1/2}}$ to improve finite-sample performance. The following metrics were used to compare the methods:

- Bias: average difference between estimates and β_0 over the Monte Carlo iterations. In all simulations $\beta_0 = 0.5$.
- Rel. Bias: relative bias, equal to bias divided by β_0.
- MSE: mean squared error of estimates over the Monte Carlo iterations.
- 95% CI Length: mean length of 95% confidence intervals.
- 95% CI CVG: coverage, i.e. proportion of Monte Carlo iterations in which the 95% confidence interval included β_0.
- Power: Proportion of Monte Carlo iterations in which the 95% confidence interval did not include 0.

Table S1: Simulation results for rough **U** and rough **A**. Metrics are bias, relative bias (bias divided by β_0), mean squared error (MSE), confidence interval (CI) length, coverage (CVG), and power. CI length, coverage, and power are computed with respect to 95% confidence intervals.

	Bias	Rel. Bias	MSE	CI Length	CVG	Power
OLS	0.499	0.997	0.273	0.170	0.005	1.000
LMM	0.474	0.947	0.232	0.329	0.000	1.000
Spline (GCV)	0.469	0.938	0.228	0.358	0.000	1.000
Spline (REML)	0.474	0.948	0.233	0.332	0.000	1.000
Spatial+	0.483	0.966	0.303	1.256	0.700	0.897
gSEM	0.472	0.944	0.291	1.248	0.703	0.905
DSR (theory)	0.452	0.905	0.215	0.425	0.005	1.000
DSR (theory, GpGp)	0.301	0.602	0.141	0.786	0.625	0.965
DSR (theory, no crossfit)	0.482	0.965	0.251	0.466	0.030	1.000
DSR	0.321	0.642	0.152	0.791	0.623	0.940
DSR (no crossfit)	0.291	0.582	0.132	0.812	0.655	0.940
DSR (spline, no crossfit)	0.332	0.663	0.147	0.708	0.527	0.985
DSR (theory, spline, no crossfit)	0.472	0.944	0.291	0.942	0.478	0.958
DSR (theory, GpGp, smooth)	**0.284**	0.568	**0.128**	0.773	0.650	0.965
DSR (smooth)	0.320	0.639	0.148	0.801	0.640	0.953

Table S2: Simulation results for rough **U** and smooth **A**. Metrics are bias, relative bias (bias divided by β_0), mean squared error (MSE), confidence interval (CI) length, coverage (CVG), and power. CI length, coverage, and power are computed with respect to 95% confidence intervals.

	Bias	Rel. Bias	MSE	CI Length	CVG	Power
OLS	0.432	0.863	0.222	0.179	0.038	1.000
LMM	0.441	0.881	0.213	0.505	0.090	1.000
Spline (GCV)	0.463	0.925	0.239	0.700	0.215	0.998
Spline (REML)	0.484	0.969	0.257	0.624	0.128	1.000
Spatial+	0.237	0.475	0.184	1.634	0.950	0.415
gSEM	0.211	0.422	0.163	1.559	0.948	0.418
DSR (theory)	0.478	0.955	0.265	0.824	0.370	0.998
DSR (theory, GpGp)	0.162	0.325	0.140	1.215	0.877	0.570
DSR (theory, no crossfit)	0.418	0.836	0.218	0.897	0.573	0.983
DSR	**0.141**	0.281	0.150	1.450	0.922	0.448
DSR (no crossfit)	0.160	0.320	**0.117**	1.216	0.895	0.568
DSR (spline, no crossfit)	0.252	0.504	0.123	0.819	0.720	0.925
DSR (theory, spline, no crossfit)	0.211	0.422	0.163	1.309	0.875	0.557
DSR (theory, GpGp, smooth)	0.165	0.329	0.137	1.215	0.900	0.578
DSR (smooth)	0.152	0.305	0.150	1.461	0.917	0.465

Table S3: Simulation results for smooth **U** and rough **A**. Metrics are bias, relative bias (bias divided by β_0), mean squared error (MSE), confidence interval (CI) length, coverage (CVG), and power. CI length, coverage, and power are computed with respect to 95% confidence intervals.

	Bias	Rel. Bias	MSE	CI Length	CVG	Power
OLS	0.402	0.804	0.195	0.172	0.040	1.000
LMM	0.179	0.358	0.038	0.295	0.340	1.000
Spline (GCV)	0.181	0.362	0.039	0.289	0.315	1.000
Spline (REML)	0.177	0.353	0.037	0.295	0.350	1.000
Spatial+	0.173	0.345	0.097	1.245	0.965	0.595
gSEM	0.169	0.338	0.095	1.238	0.973	0.603
DSR (theory)	0.182	0.365	0.042	0.409	0.610	1.000
DSR (theory, GpGp)	0.027	0.054	0.042	0.756	0.935	0.762
DSR (theory, no crossfit)	0.236	0.472	0.072	0.466	0.488	1.000
DSR	0.033	0.066	0.041	0.775	0.968	0.780
DSR (no crossfit)	0.041	0.082	0.048	0.830	0.950	0.713
DSR (spline, no crossfit)	0.038	0.077	**0.036**	0.713	0.950	0.830
DSR (theory, spline, no crossfit)	0.169	0.338	0.095	0.958	0.885	0.770
DSR (theory, GpGp, smooth)	**0.004**	0.007	0.042	0.743	0.940	0.713
DSR (smooth)	0.023	0.046	0.045	0.772	0.938	0.745

Table S4: Simulation results for smooth **U** and smooth **A**. Metrics are bias, relative bias (bias divided by β_0), mean squared error (MSE), confidence interval (CI) length, coverage (CVG), and power. CI length, coverage, and power are computed with respect to 95% confidence intervals.

	Bias	Rel. Bias	MSE	CI Length	CVG	Power
OLS	0.498	0.997	0.291	0.174	0.028	1.000
LMM	0.434	0.869	0.206	0.461	0.065	1.000
Spline (GCV)	0.435	0.870	0.208	0.463	0.075	1.000
Spline (REML)	0.431	0.862	0.204	0.477	0.088	1.000
Spatial+	0.210	0.419	0.163	1.619	0.950	0.398
gSEM	0.194	0.388	0.151	1.552	0.950	0.412
DSR (theory)	0.431	0.862	0.216	0.790	0.448	1.000
DSR (theory, GpGp)	0.046	0.093	0.098	1.174	0.938	0.462
DSR (theory, no crossfit)	0.386	0.772	0.189	0.884	0.618	0.983
DSR	0.018	0.036	0.121	1.458	0.963	0.345
DSR (no crossfit)	0.047	0.094	0.093	1.295	0.968	0.415
DSR (spline, no crossfit)	0.180	0.359	**0.080**	0.840	0.828	0.858
DSR (theory, spline, no crossfit)	0.194	0.388	0.151	1.320	0.887	0.545
DSR (theory, GpGp, smooth)	0.048	0.096	0.095	1.175	0.930	0.452
DSR (smooth)	**0.016**	0.032	0.114	1.448	0.970	0.332

Table S5: Simulation results for middle-out heteroskedastic errors. Metrics are bias, relative bias (bias divided by β_0), mean squared error (MSE), confidence interval (CI) length, coverage (CVG), and power. CI length, coverage, and power are computed with respect to 95% confidence intervals.

	Bias	Rel. Bias	MSE	CI Length	CVG	Power
OLS	0.165	0.329	0.036	0.106	0.102	1.000
LMM	0.151	0.301	**0.027**	0.236	0.310	1.000
Spline (GCV)	0.151	0.302	0.028	0.266	0.428	1.000
Spline (REML)	0.152	0.304	0.028	0.252	0.380	1.000
Spatial+	0.162	0.325	0.087	1.139	0.948	0.632
gSEM	0.149	0.298	0.081	1.089	0.950	0.637
DSR (theory)	0.220	0.441	0.063	0.550	0.667	1.000
DSR (theory, GpGp)	0.020	0.039	0.049	0.824	0.945	0.680
DSR (theory, no crossfit)	0.210	0.420	0.067	0.621	0.738	0.995
DSR	0.010	0.020	0.061	0.970	0.975	0.565
DSR (no crossfit)	0.022	0.045	0.044	0.847	0.958	0.665
DSR (spline, no crossfit)	0.062	0.123	**0.027**	0.585	0.938	0.940
DSR (theory, spline, no crossfit)	0.149	0.298	0.081	0.934	0.900	0.760
DSR (theory, GpGp, smooth)	0.020	0.041	0.049	0.823	0.940	0.680
DSR (smooth)	**0.007**	0.014	0.063	0.980	0.965	0.555

Table S6: Simulation results for cubed confounder. Metrics are bias, relative bias (bias divided by β_0), mean squared error (MSE), confidence interval (CI) length, coverage (CVG), and power. CI length, coverage, and power are computed with respect to 95% confidence intervals.

	Bias	Rel. Bias	MSE	CI Length	CVG	Power
OLS	1.449	2.899	3.164	0.426	0.025	1.000
LMM	0.465	0.929	0.292	1.029	0.557	0.925
Spline (GCV)	0.558	1.115	0.398	1.021	0.455	0.970
Spline (REML)	0.466	0.931	0.294	1.069	0.598	0.927
Spatial+	0.114	0.228	0.141	1.638	0.980	0.285
gSEM	0.077	0.155	0.119	1.552	0.978	0.282
DSR (theory)	0.935	1.869	1.175	1.163	0.192	0.990
DSR (theory, GpGp)	0.053	0.107	0.136	1.260	0.915	0.402
DSR (theory, no crossfit)	0.749	1.498	0.777	1.163	0.338	0.983
DSR	0.009	0.018	0.150	1.602	0.953	0.280
DSR (no crossfit)	0.089	0.179	**0.112**	1.203	0.900	0.488
DSR (spline, no crossfit)	0.263	0.526	0.158	0.798	0.680	0.882
DSR (theory, spline, no crossfit)	0.077	0.155	0.119	1.254	0.925	0.448
DSR (theory, GpGp, smooth)	0.059	0.117	0.134	1.260	0.912	0.438
DSR (smooth)	**0.001**	0.002	0.159	1.636	0.955	0.288

Table S7: Simulation results for gamma-distributed errors. Metrics are bias, relative bias (bias divided by β_0), mean squared error (MSE), confidence interval (CI) length, coverage (CVG), and power. CI length, coverage, and power are computed with respect to 95% confidence intervals.

	Bias	Rel. Bias	MSE	CI Length	CVG	Power
OLS	0.416	0.832	0.210	0.166	0.055	1.000
LMM	0.367	0.733	0.151	0.429	0.108	1.000
Spline (GCV)	0.364	0.728	0.151	0.452	0.148	1.000
Spline (REML)	0.364	0.729	0.151	0.450	0.142	1.000
Spatial+	0.199	0.398	0.158	1.619	0.963	0.385
gSEM	0.184	0.368	0.148	1.552	0.960	0.400
DSR (theory)	0.390	0.780	0.183	0.790	0.500	0.995
DSR (theory, GpGp)	0.034	0.068	0.094	1.171	0.953	0.448
DSR (theory, no crossfit)	0.360	0.720	0.171	0.888	0.665	0.975
DSR	0.008	0.016	0.119	1.435	0.965	0.335
DSR (no crossfit)	0.038	0.075	0.094	1.278	0.965	0.410
DSR (spline, no crossfit)	0.150	0.299	**0.072**	0.834	0.858	0.835
DSR (theory, spline, no crossfit)	0.184	0.368	0.148	1.323	0.890	0.530
DSR (theory, GpGp, smooth)	0.035	0.070	0.093	1.173	0.943	0.450
DSR (smooth)	**-0.002**	-0.004	0.118	1.436	0.973	0.340

Table S8: Simulation results for east-west heteroskedastic errors. Metrics are bias, relative bias (bias divided by β_0), mean squared error (MSE), confidence interval (CI) length, coverage (CVG), and power. CI length, coverage, and power are computed with respect to 95% confidence intervals.

	Bias	Rel. Bias	MSE	CI Length	CVG	Power
OLS	0.250	0.499	0.078	0.150	0.072	1.000
LMM	0.230	0.461	0.062	0.331	0.230	1.000
Spline (GCV)	0.230	0.461	0.063	0.369	0.320	1.000
Spline (REML)	0.232	0.464	0.063	0.351	0.282	1.000
Spatial+	0.195	0.389	0.156	1.618	0.960	0.382
gSEM	0.182	0.365	0.147	1.552	0.948	0.388
DSR (theory)	0.350	0.700	0.151	0.783	0.603	0.993
DSR (theory, GpGp)	0.026	0.052	0.094	1.166	0.938	0.445
DSR (theory, no crossfit)	0.331	0.662	0.156	0.887	0.703	0.970
DSR	**0.006**	0.013	0.109	1.368	0.968	0.352
DSR (no crossfit)	0.024	0.048	0.088	1.232	0.965	0.420
DSR (spline, no crossfit)	0.092	0.184	**0.053**	0.832	0.907	0.780
DSR (theory, spline, no crossfit)	0.182	0.365	0.147	1.333	0.895	0.520
DSR (theory, GpGp, smooth)	0.023	0.046	0.089	1.166	0.960	0.432
DSR (smooth)	0.007	0.013	0.113	1.395	0.973	0.330

Table S9: Simulation results for $\sigma_A^2 = 1$. Metrics are bias, relative bias (bias divided by β_0), mean squared error (MSE), confidence interval (CI) length, coverage (CVG), and power. CI length, coverage, and power are computed with respect to 95% confidence intervals. Note that Shift (BART) results are included, without variance estimates.

	Bias	Rel. Bias	MSE	CI Length	CVG	Power
OLS	0.232	0.464	0.065	0.121	0.070	1
LMM	0.025	0.050	0.002	0.124	0.855	1
Spline (GCV)	0.027	0.054	0.002	0.124	0.858	1
Spline (REML)	0.024	0.049	0.002	0.124	0.870	1
Spatial+	0.020	0.041	0.001	0.161	0.978	1
gSEM	0.003	0.005	0.001	0.165	0.988	1
Shift (BART)	**-0.002**	-0.005	0.002	-	-	-
DSR (theory)	0.012	0.023	0.001	0.124	0.930	1
DSR (theory, GpGp)	0.008	0.015	0.001	0.124	0.940	1
DSR (theory, no crossfit)	0.007	0.014	0.001	0.123	0.938	1
DSR	0.009	0.018	0.001	0.123	0.940	1
DSR (no crossfit)	0.010	0.020	0.001	0.115	0.922	1
DSR (spline, no crossfit)	0.009	0.018	0.001	0.115	0.920	1
DSR (theory, spline, no crossfit)	0.003	0.005	**0.001**	0.123	0.945	1
DSR (theory, GpGp, smooth)	0.008	0.016	0.001	0.124	0.948	1
DSR (smooth)	0.010	0.019	0.001	0.123	0.948	1

Table S10: Simulation results for very rough, exponential spatial processes generating **U** and **A**. Metrics are bias, relative bias (bias divided by β_0), mean squared error (MSE), confidence interval (CI) length, coverage (CVG), and power. CI length, coverage, and power are computed with respect to 95% confidence intervals.

	Bias	Rel. Bias	MSE	CI Length	CVG	Power
OLS	0.501	1.003	0.265	0.168	0.000	1
LMM	0.491	0.981	0.245	0.237	0.000	1
Spline (GCV)	0.489	0.978	0.243	0.253	0.000	1
Spline (REML)	0.492	0.984	0.246	0.234	0.000	1
Spatial+	0.608	1.216	0.380	0.483	0.000	1
gSEM	0.582	1.165	0.350	0.481	0.002	1
DSR (theory)	0.508	1.017	0.263	0.274	0.000	1
DSR (theory, GpGp)	0.473	0.947	0.233	0.328	0.002	1
DSR (theory, no crossfit)	0.554	1.108	0.319	0.297	0.000	1
DSR	0.474	0.947	0.232	0.324	0.000	1
DSR (no crossfit)	0.478	0.956	0.236	0.282	0.002	1
DSR (spline, no crossfit)	0.478	0.956	0.235	0.299	0.000	1
DSR (theory, spline, no crossfit)	0.582	1.165	0.350	0.359	0.000	1
DSR (theory, GpGp, smooth)	**0.446**	0.893	**0.207**	0.321	0.000	1
DSR (smooth)	0.478	0.957	0.236	0.322	0.000	1

Table S11: Simulation results for spatial locations located on a regular grid. Metrics are bias, relative bias (bias divided by β_0), mean squared error (MSE), confidence interval (CI) length, coverage (CVG), and power. CI length, coverage, and power are computed with respect to 95% confidence intervals.

	Bias	Rel. Bias	MSE	CI Length	CVG	Power
OLS	0.488	0.975	0.277	0.171	0.030	1.000
LMM	0.431	0.863	0.205	0.442	0.075	1.000
Spline (GCV)	0.430	0.859	0.205	0.444	0.085	1.000
Spline (REML)	0.428	0.855	0.203	0.455	0.095	1.000
Spatial+	0.215	0.430	0.167	1.596	0.945	0.390
gSEM	0.199	0.398	0.155	1.529	0.940	0.415
DSR (theory)	0.435	0.869	0.221	0.767	0.378	1.000
DSR (theory, GpGp)	0.046	0.092	0.095	1.152	0.943	0.455
DSR (theory, no crossfit)	0.407	0.815	0.212	0.866	0.560	0.993
DSR	**0.006**	0.013	0.116	1.446	0.965	0.325
DSR (no crossfit)	0.025	0.050	0.101	1.373	0.968	0.328
DSR (spline, no crossfit)	0.180	0.360	**0.081**	0.817	0.828	0.853
DSR (theory, spline, no crossfit)	0.199	0.398	0.155	1.300	0.887	0.550
DSR (theory, GpGp, smooth)	0.057	0.114	0.098	1.153	0.925	0.460
DSR (smooth)	0.013	0.027	0.116	1.454	0.963	0.318

Table S12: Simulation results for deterministic latent functions of space g_0 and m_0 such that $g_0 = m_0$. Metrics are bias, relative bias (bias divided by β_0), mean squared error (MSE), confidence interval (CI) length, coverage (CVG), and power. CI length, coverage, and power are computed with respect to 95% confidence intervals.

	Bias	Rel. Bias	MSE	CI Length	CVG	Power
OLS	0.959	1.919	0.924	0.244	0.000	1.000
LMM	0.957	1.913	0.919	0.252	0.000	1.000
Spline (GCV)	0.956	1.913	0.919	0.252	0.000	1.000
Spline (REML)	0.958	1.917	0.922	0.247	0.000	1.000
Spatial+	0.227	0.454	0.170	1.600	0.953	0.428
gSEM	0.208	0.416	0.156	1.521	0.938	0.450
DSR (theory)	0.576	1.152	0.388	0.997	0.350	0.990
DSR (theory, GpGp)	0.045	0.090	0.109	1.209	0.920	0.458
DSR (theory, no crossfit)	0.467	0.935	0.300	1.087	0.588	0.932
DSR	0.141	0.281	**0.102**	1.227	0.925	0.568
DSR (no crossfit)	0.301	0.602	0.159	0.967	0.745	0.858
DSR (spline, no crossfit)	0.371	0.743	0.181	0.874	0.603	0.963
DSR (theory, spline, no crossfit)	0.208	0.416	0.156	1.304	0.892	0.580
DSR (theory, GpGp, smooth)	**0.051**	0.102	0.106	1.210	0.935	0.448
DSR (smooth)	0.146	0.292	**0.102**	1.225	0.938	0.580

Table S13: Simulation results for deterministic latent functions of space g_0 and m_0 such that $g_0 \neq m_0$. Metrics are bias, relative bias (bias divided by β_0), mean squared error (MSE), confidence interval (CI) length, coverage (CVG), and power. CI length, coverage, and power are computed with respect to 95% confidence intervals.

	Bias	Rel. Bias	MSE	CI Length	CVG	Power
OLS	0.987	1.974	0.979	0.271	0.000	1.000
LMM	0.873	1.746	0.769	0.511	0.000	1.000
Spline (GCV)	0.844	1.688	0.722	0.541	0.000	1.000
Spline (REML)	0.849	1.698	0.729	0.538	0.000	1.000
Spatial+	0.195	0.390	0.156	1.600	0.965	0.395
gSEM	0.176	0.352	0.143	1.521	0.960	0.418
DSR (theory)	0.529	1.058	0.334	1.003	0.430	0.985
DSR (theory, GpGp)	0.037	0.074	0.104	1.213	0.945	0.408
DSR (theory, no crossfit)	0.394	0.787	0.226	1.087	0.713	0.910
DSR	0.085	0.171	**0.099**	1.254	0.945	0.458
DSR (no crossfit)	0.238	0.477	0.120	0.993	0.812	0.818
DSR (spline, no crossfit)	0.333	0.666	0.161	0.836	0.618	0.958
DSR (theory, spline, no crossfit)	0.176	0.352	0.143	1.298	0.892	0.542
DSR (theory, GpGp, smooth)	**0.043**	0.087	0.104	1.213	0.930	0.425
DSR (smooth)	0.087	0.173	0.101	1.252	0.948	0.465

S6 Proof of Theorem 1

Theorem 1 follows from an extension of Theorem 4.1 from (Chernozhukov et al., 2018), and Theorems 3.3 and 3.6 from (Eberts and Steinwart, 2013). This section extends Theorem 4.1 from (Chernozhukov et al., 2018) to the case of a vector treatment variable, and then verifies that Assumptions A1-A6 satisfy the necessary conditions to apply these results.

S6.1 DML with partially linear model

In this section we extend Theorem 4.1 from (Chernozhukov et al., 2018), which analyzes the partially linear model with a scalar treatment, to a vector treatment. The extension essentially follows the proof of Theorem 4.1 from (Chernozhukov et al., 2018) with slight changes.

We use “DML2”, which is Definition 3.2 from Chernozhukov et al. (2018), where rather than aggregating K different estimates from K different folds, cross-fitting on the K folds is performed followed by estimation of $\boldsymbol{\beta}_0$ using the combined cross-fitted estimates.

The assumed model is:

$$\begin{aligned} \mathbf{Y} &= \mathbf{X}\boldsymbol{\beta} + g_0(\mathbf{S}) + \mathbf{U} \\ \mathbf{X}_j &= m_{0j}(\mathbf{S}) + \mathbf{V}_j \end{aligned} \tag{11}$$

with notation and definitions as in the main paper. Nuisance parameters and estimates η are assumed to be in T, a convex subset of some normed vector space.

The “practical” DSR estimator uses the score function:

$$\psi(\mathbf{W};\boldsymbol{\beta},\eta) := \{Y - g(\mathbf{S}) - \mathbf{X}^T\boldsymbol{\beta}\}(\mathbf{X} - m(\mathbf{S})) \tag{12}$$

However, for theory, we focus on the score function:

$$\psi(\mathbf{W};\boldsymbol{\beta},\eta) := \{Y - h(\mathbf{S}) - (\mathbf{X} - m(\mathbf{S}))^T\boldsymbol{\beta}\}(\mathbf{X} - m(\mathbf{S})) \tag{13}$$

Assumption 1 consists of those of Assumption 4.1 from Chernozhukov et al. (2018) but expanded to encompass the case $p > 1$, the length of $\boldsymbol{\beta}_0$, and with some other slight changes for theoretical convenience. Let $\{\delta_n\}$ and $\{\Delta_n\}$ be sequences of positive constants converging to 0. Let c, C, and q be fixed strictly positive constants such that $q > 4$, and let $K \geqslant 2$ be a fixed integer. For any $\eta = (\ell_1, \ell_2, ..., \ell_m)$ for any positive integer m such that ℓ_1, ..., ℓ_m are functions mapping $\mathcal{S}$ to $\mathbb{R}$, denote $\|\eta\|_{P,q} = \max_{1,...,m}\{\|\ell_1\|_{P,q}, ..., \|\ell_m\|_{P,q}\}$.

Assumption 1 (Regularity Conditions for partially linear regression model) *Let $\mathcal{P}$ be the collection of probability laws P for $\mathbf{W} = (Y, \mathbf{X}, \mathbf{S})$ such that*

a) Model (11) holds,

b) $\|V_1\|_{P,q}, ..., \|V_p\|_{P,q} \leqslant C$ and $\|\boldsymbol{\beta}_0\|_\infty \leqslant C$,

c) The eigenvalues of the matrix $E[U^2\mathbf{V}\mathbf{V}^T]$ are greater than or equal to c^2 and the matrix $E[\mathbf{V}\mathbf{V}^T]$ has singular values at least c and no greater than C,

d) $\|E[U^2|\mathbf{S}]\|_{P,\infty} \leqslant C$ and $\|E[V_j^2|\mathbf{S}]\|_{P,\infty} \leqslant C$ for $j = 1, ..., p$,

e) Given a random subset I of $[n]$ of size n/K, the nuisance parameter estimator $\widehat{\eta}_0 = \widehat{\eta}_0((\mathbf{W}_i)_{i\in I^C})$ obey the following conditions for all $n/K \geqslant 1$: with P-probability no less than $1 - \Delta_n$,

$$\|\widehat{\eta}_0 - \eta_0\|_{P,\infty} \leqslant C, \|\widehat{\eta}_0 - \eta_0\|_{P,2} \leqslant \delta_n,$$

and for the score ψ in (13), where $\widehat{\eta} = (\widehat{\ell}_0, \widehat{m}_{10}, ..., \widehat{m}_{p0})$,

$$\|\widehat{m}_{j0} - m_{j0}\|_{P,2} \times (\|\widehat{m}_{j0} - m_{j0}\|_{P,2} + \|\widehat{\ell}_0 - \ell_0\|_{P,2}) \leqslant \delta_n n^{-1/2} \text{ for } j = 1, ..., p.$$

The following lemma is Theorem 4.1 from Chernozhukov et al. (2018) but expanded to the case $p > 1$.

Lemma 1 (DML inference in the partially linear regression model with $p > 1$) *Suppose that Assumption 1 holds. Then the DML2 estimator constructed in Definition 3.2 of Chernozhukov et al. (2018) using the score (13) obeys*

$$\boldsymbol{\Sigma}^{-1/2}\sqrt{n}(\widehat{\boldsymbol{\beta}}_0 - \boldsymbol{\beta}_0) \xrightarrow{D} N(\mathbf{0}, \mathbf{I}_p),$$

uniformly over $P \in \mathcal{P}$, where $\boldsymbol{\Sigma} = [E(\mathbf{V}\mathbf{V}^T)]^{-1}E(U^2\mathbf{V}\mathbf{V}^T)[E(\mathbf{V}\mathbf{V}^T)]^{-1}$. The result continues to hold if $\boldsymbol{\Sigma}$ is replaced by $\widehat{\boldsymbol{\Sigma}}$ from Theorem 3.2 from Chernozhukov et al. (2018).

The proof for Lemma 1 follows the proof of Theorem 4.1 in Chernozhukov et al. (2018) closely, only needing to add steps to deal with the vector-valued ψ and matrix-valued ψ^a, and changing some assumptions slightly for convenience. The proof verifies Assumptions 3.1 and 3.2 from Chernozhukov et al. (2018), from which the conclusion of Lemma 1 follows from Theorems 3.1 and 3.2 from Chernozhukov et al. (2018).

Proof 1 (Proof of Lemma 1) *Observe that the score (13) is linear in $\boldsymbol{\beta}$:*

$$\psi(\mathbf{W}; \boldsymbol{\beta}, \eta) = \{Y - \ell(\mathbf{S}) - (\mathbf{X} - \mathbf{m}(\mathbf{S}))^T\boldsymbol{\beta}\}(\mathbf{X} - \mathbf{m}(\mathbf{S})) = \psi^a(\mathbf{W}; \eta)\boldsymbol{\beta} + \psi^b(\mathbf{W}; \eta),$$
$$\psi^a(\mathbf{W}; \eta) = -(\mathbf{X} - \mathbf{m}(\mathbf{S}))(\mathbf{X} - \mathbf{m}(\mathbf{S}))^T, \quad \psi^b(\mathbf{W}; \eta) = (Y - \ell(\mathbf{S}))(\mathbf{X} - \mathbf{m}(\mathbf{S}))$$

Therefore, it is sufficient to verify Assumptions 3.1 and 3.2 from Chernozhukov et al. (2018). Let $\mathcal{T}_n$ be the set of all $\eta = (\ell, m_1, ..., m_p)$ consisting of $P-$square-integrable functions $\ell, m_1, ..., m_p$ such that

$$\|\widehat{\eta}_0 - \eta_0\|_{P,q} \leqslant C, \|\widehat{\eta}_0 - \eta_0\|_{P,2} \leqslant \delta_n,$$
$$\|\widehat{m}_{j0} - m_{j0}\|_{P,2} \times (\|\widehat{m}_{j0} - m_{j0}\|_{P,2} + \|\widehat{\ell}_0 - \ell_0\|_{P,2}) \leqslant \delta_n n^{-1/2}.$$

We replace the constant q and the sequence $\{\delta_n\}$ in Assumptions 3.1 and 3.2 from Chernozhukov et al. (2018) by $q/2$ and $\{\delta_n'\}$, with $\delta_n' = (2p^2C + \sqrt{C}p + \sqrt{pC} + pC + pC\sqrt{pC} + \sqrt{p}pC^2 + 4\sqrt{p})(\delta_n \vee n^{-[(1-4/q)\wedge(1/2)]})$ for all n. As in Chernozhukov et al. (2018), we use five steps.

Step 1. *Verify Neyman orthogonality. Note that $E[\psi(\mathbf{W}; \boldsymbol{\beta}_0, \eta_0)] = 0$ by the definitions of $\boldsymbol{\beta}_0, \eta_0$. For any $\eta \in \mathcal{T}_n$, the Gateaux derivative in the direction $\eta - \eta_0$ is given by, for $r = 0$, (see derivation in Step 5):*

$$\begin{aligned}\partial_\eta E[\psi(\mathbf{W}; \boldsymbol{\beta}_0, \eta_0)][\eta - \eta_0] = &-E[(Y - \ell_0(\mathbf{S}))(\mathbf{m}(\mathbf{S}) - \mathbf{m}_0(\mathbf{S}))] - E[(\ell(\mathbf{S}) - \ell_0(\mathbf{S}))(\mathbf{D} - \mathbf{m}_0(\mathbf{S}))] \\ &+ E[(\mathbf{D} - \mathbf{m}_0(\mathbf{S}))(\mathbf{m}(\mathbf{S}) - \mathbf{m}_0(\mathbf{S}))^T\boldsymbol{\beta}_0] \\ &+ E[(\mathbf{m}(\mathbf{S}) - \mathbf{m}_0(\mathbf{S}))(\mathbf{D} - \mathbf{m}_0(\mathbf{S}))^T\boldsymbol{\beta}_0].\end{aligned}$$

By the law of iterated expectation, and since $\mathbf{V} = \mathbf{X} - \mathbf{m}_0(\mathbf{S})$ *and* $U = Y - \ell_0(\mathbf{S})$*:*

$$\begin{aligned}\partial_\eta E[\psi(\mathbf{W};\boldsymbol{\beta}_0,\eta_0)][\eta-\eta_0] &= E\{E[U \times (\mathbf{m}(\mathbf{S}) - \mathbf{m}_0(\mathbf{S}))|\mathbf{X},\mathbf{S}]\} \\ &\quad - E\{E[(\ell(\mathbf{S}) - \ell_0(\mathbf{S}))\mathbf{V}|\mathbf{S}]\} \\ &\quad + E\{E[(\mathbf{m}(\mathbf{S}) - \mathbf{m}_0(\mathbf{S}))\mathbf{V}^T\boldsymbol{\beta}_0|\mathbf{S}]\} \\ &\quad + E\{E[\mathbf{V}(\mathbf{m}(\mathbf{S}) - \mathbf{m}_0(\mathbf{S}))^T\boldsymbol{\beta}_0|\mathbf{S}]\},\end{aligned}$$

which is equal to 0 since $E[\mathbf{V}|\mathbf{S}] = \mathbf{0}$ *and* $E[U|\mathbf{X},\mathbf{S}] = 0$*. This gives Assumption 3.1(d) from Chernozhukov et al. (2018) with* $\lambda_n = 0$*.*

Step 2. *By definition,*

$$\begin{aligned}J_0 &= E[\psi^a(\mathbf{W};\eta_0)] \\ &= E[-(\mathbf{X} - \mathbf{m}_0(\mathbf{S}))(\mathbf{X} - \mathbf{m}_0(\mathbf{S}))^T] \\ &= E[-\mathbf{V}\mathbf{V}^T]\end{aligned}$$

By assumption 1(c), the singular values of this matrix are between c_0 *and* C*. This satisfies Assumption 3.1 (e) from Chernozhukov et al. (2018). Since the map* $\eta \mapsto E[\psi(\mathbf{W};\boldsymbol{\beta}_0,\eta)]$ *is twice Gateux-differentiable in* T*, this completes verification of Assumption 3.1 from Chernozhukov et al. (2018).*

Step 3. *Assumption 3.2(a) from Chernozhukov et al. (2018) holds by construction of* $\mathcal{T}_n$ *and Assumption 1(e). In addition,* $\psi(\mathbf{W};\boldsymbol{\beta}_0,\eta_0) = U\mathbf{V}$*, so the eigenvalues of the matrix*

$$E[\psi(\mathbf{W};\boldsymbol{\beta}_0,\eta_0)\psi(\mathbf{W};\boldsymbol{\beta}_0,\eta_0)^T] = E[U^2\mathbf{V}\mathbf{V}^T]$$

are bounded below by c_0 *by Assumption 1(c). This verifies Assumption 3.2(d) from Chernozhukov et al. (2018).*

Step 4. *Next, we verify Assumption 3.2(b) from Chernozhukov et al. (2018). For any* $\eta = (\ell, \mathbf{m}) \in \mathcal{T}_n$*,*

$$\begin{aligned}m_n' &= E[\|\psi^a(\mathbf{S};\eta)\|^{q/2}]^{2/q} \\ &= \big\|\|(\mathbf{X} - \mathbf{m}(\mathbf{S}))(\mathbf{X} - \mathbf{m}(\mathbf{S}))^T\|\big\|_{P,q/2} \\ &\leqslant \big\|\|(\mathbf{X} - \mathbf{m}_0(\mathbf{S}))(\mathbf{X} - \mathbf{m}_0(\mathbf{S}))^T\| + \|(\mathbf{X} - \mathbf{m}_0(\mathbf{S}))(\mathbf{m}_0(\mathbf{S}) - \mathbf{m}(\mathbf{S}))^T\| \\ &+ \|(\mathbf{m}_0(\mathbf{S}) - \mathbf{m}(\mathbf{S}))(\mathbf{X} - \mathbf{m}_0(\mathbf{S}))^T\| + \|(\mathbf{m}_0(\mathbf{S}) - \mathbf{m}(\mathbf{S}))(\mathbf{m}_0(\mathbf{S}) - \mathbf{m}(\mathbf{S}))^T\|\big\|_{P,q/2} \\ &= \big\|\|\mathbf{V}\mathbf{V}^T\| + 2\|\mathbf{V}(\mathbf{m}_0(\mathbf{S}) - \mathbf{m}(\mathbf{S}))^T\| + \|(\mathbf{m}_0(\mathbf{S}) - \mathbf{m}(\mathbf{S}))(\mathbf{m}_0(\mathbf{S}) - \mathbf{m}(\mathbf{S}))^T\|\big\|_{P,q/2} \\ &\leqslant \big\|\|\mathbf{V}\mathbf{V}^T\|\big\|_{P,q/2} + 2\big\|\|\mathbf{V}(\mathbf{m}_0(\mathbf{S}) - \mathbf{m}(\mathbf{S}))^T\|\big\|_{P,q/2} + \big\|\|(\mathbf{m}_0(\mathbf{S}) - \mathbf{m}(\mathbf{S}))(\mathbf{m}_0(\mathbf{S}) - \mathbf{m}(\mathbf{S}))^T\|\big\|_{P,q/2} \\ &= \big\|\|\mathbf{V}\|^2\big\|_{P,q/2} + 2\big\|\|\mathbf{V}\| \times \|(\mathbf{m}_0(\mathbf{S}) - \mathbf{m}(\mathbf{S}))\|\big\|_{P,q/2} + \big\|\|(\mathbf{m}_0(\mathbf{S}) - \mathbf{m}(\mathbf{S}))\|^2\big\|_{P,q/2} \\ &\leqslant \big\|\|\mathbf{V}\|\big\|_{P,q}^2 + 2\big\|\|\mathbf{V}\|\big\|_{P,q} \times \big\|\|\mathbf{m}_0(\mathbf{S}) - \mathbf{m}(\mathbf{S})\|\big\|_{P,q} + \big\|\|\mathbf{m}_0(\mathbf{S}) - \mathbf{m}(\mathbf{S})\|\big\|_{P,q}^2,\end{aligned}$$

by the triangle inequality for the $\|\cdot\|$*-norm, the triangle inequality for the* $\|\cdot\|_{P,q/2}$*-norm, the fact that for vectors* $\mathbf{u}$ *and* $\mathbf{v}$*,* $\|\mathbf{u}\mathbf{v}^T\| = \|\mathbf{u}\|\|\mathbf{v}\|$*, and by the Cauchy-Schwarz inequality. Note that for a random vector* $\mathbf{Z}$ *with p elements, if* $\|Z_j\|_{P,q} < C$ *for* $j = 1, ..., p$*, then* $\|\|\mathbf{Z}\|\|_{P,q} \leqslant \|\|\mathbf{Z}\|_1\|_{P,q} \leqslant \|Z_1\|_{P,q} + ... + \|Z_p\|_{P,q} \leqslant pC$*, by the fact that* $\|\mathbf{Z}\| \leqslant \|\mathbf{Z}\|_1$ *and monotonicity of expectation, then the triangle inequality, then the assumption that* $\|Z_j\|_{P,q} < C$ *for* $j = 1, ..., p$*. By assumption* $\|V_1\|_{P,q} + ... + \|V_p\|_{P,q} \leqslant C$*. Also, for* $\eta \in \mathcal{T}_n$*,* $\|\eta_0 - \eta\|_{P,q} < C$*. Therefore we have that:*

$$\begin{aligned}m_n' &\leqslant \|\|\mathbf{V}\|\|_{P,q}^2 + 2\|\|\mathbf{V}\|\|_{P,q} \times \|\|\mathbf{m}_0(\mathbf{S}) - \mathbf{m}(\mathbf{S})\|\|_{P,q} + \|\|\mathbf{m}_0(\mathbf{S}) - \mathbf{m}(\mathbf{S})\|\|_{P,q}^2 \\ &\leqslant (pC)^2 + 2(pC)(pC) + (pC)^2 = 4p^2C^2\end{aligned}$$

bounding m_n' *as desired.*

Next we address m_n. Note that by assumption, $\|\boldsymbol{\beta}_0\| < \sqrt{p}C$ since $\|\beta_0\|_\infty < C$. Note also that $Y - \ell_0(\mathbf{S}) = U + \mathbf{V}^T\boldsymbol{\beta}_0$.

$$
\begin{aligned}
m_n &= (E[\|\psi(\mathbf{W};\boldsymbol{\beta}_0,\eta)\|^{q/2}])^{2/q} \\
&= \big\|\|\psi(\mathbf{W};\boldsymbol{\beta}_0,\eta)\|\big\|_{P,q/2} \\
&= \big\|\|U\mathbf{V} + U(\mathbf{m}_0(\mathbf{S}) - \mathbf{m}(\mathbf{S})) + (\ell_0(\mathbf{S}) - \ell(\mathbf{S}))\mathbf{V} \\
&\qquad + (\ell_0(\mathbf{S}) - \ell(\mathbf{S}))(\mathbf{m}_0(\mathbf{S}) - \mathbf{m}(\mathbf{S})) \\
&\qquad - (\mathbf{m}_0(\mathbf{S}) - \mathbf{m}(\mathbf{S}))^T\boldsymbol{\beta}_0\mathbf{V} - (\mathbf{m}_0(\mathbf{S}) - \mathbf{m}(\mathbf{S}))(\mathbf{m}_0(\mathbf{S}) - \mathbf{m}(\mathbf{S}))^T\boldsymbol{\beta}_0\|\big\|_{P,q/2} \\
&\leqslant \big\|\|U\mathbf{V}\| + \|U(\mathbf{m}_0(\mathbf{S}) - \mathbf{m}(\mathbf{S}))\| + \|(\ell_0(\mathbf{S}) - \ell(\mathbf{S}))\mathbf{V}\| + \|(\ell_0(\mathbf{S}) - \ell(\mathbf{S}))(\mathbf{m}_0(\mathbf{S}) - \mathbf{m}(\mathbf{S}))\| \\
&\qquad + \|(\mathbf{m}_0(\mathbf{S}) - \mathbf{m}(\mathbf{S}))^T\boldsymbol{\beta}_0\mathbf{V}\| + \|(\mathbf{m}_0(\mathbf{S}) - \mathbf{m}(\mathbf{S}))(\mathbf{m}_0(\mathbf{S}) - \mathbf{m}(\mathbf{S}))^T\boldsymbol{\beta}_0\|\big\|_{P,q/2} \\
&\leqslant \big\|\|U\mathbf{V}\|\big\|_{P,q/2} + \big\|\|U(\mathbf{m}_0(\mathbf{S}) - \mathbf{m}(\mathbf{S}))\|\big\|_{P,q/2} + \big\|\|(\ell_0(\mathbf{S}) - \ell(\mathbf{S}))\mathbf{V}\|\big\|_{P,q/2} \\
&\qquad + \big\|\|(\ell_0(\mathbf{S}) - \ell(\mathbf{S}))(\mathbf{m}_0(\mathbf{S}) - \mathbf{m}(\mathbf{S}))\|\big\|_{P,q/2} \\
&\qquad + \big\|\|\mathbf{V}(\mathbf{m}_0(\mathbf{S}) - \mathbf{m}(\mathbf{S}))^T\boldsymbol{\beta}_0\|\big\|_{P,q/2} + \big\|\|(\mathbf{m}_0(\mathbf{S}) - \mathbf{m}(\mathbf{S}))(\mathbf{m}_0(\mathbf{S}) - \mathbf{m}(\mathbf{S}))^T\boldsymbol{\beta}_0\|\big\|_{P,q/2} \\
&\leqslant \|U\|_{P,q}\big\|\|\mathbf{V}\|\big\|_{P,q} + \|U\|_{P,q}\big\|\|\mathbf{m}_0(\mathbf{S}) - \mathbf{m}(\mathbf{S})\|\big\|_{P,q} + \|\ell_0(\mathbf{S}) - \ell(\mathbf{S})\|_{P,q}\big\|\|\mathbf{V}\|\big\|_{P,q} \\
&\qquad + \|\ell_0(\mathbf{S}) - \ell(\mathbf{S})\|_{P,q}\big\|\|\mathbf{m}_0(\mathbf{S}) - \mathbf{m}(\mathbf{S})\|\big\|_{P,q} \\
&\qquad + \big\|\|\mathbf{V}\|\big\|_{P,q}\big\|\|\mathbf{m}_0(\mathbf{S}) - \mathbf{m}(\mathbf{S})\|\big\|_{P,q}\|\boldsymbol{\beta}_0\| + \big\|\|\mathbf{m}_0(\mathbf{S}) - \mathbf{m}(\mathbf{S})\|\big\|^2_{P,q}\|\boldsymbol{\beta}_0\| \\
&\leqslant pC^2 + pC^2 + pC^2 + pC^2 + p^2C^3 + p^2C^3 \\
&= 4pC^2 + 2p^2C^3,
\end{aligned}
$$

bounding m_n as desired. The first two inequalities are due to the triangle inequality; the third inequality due to Cauchy-Schwartz; and the final inequality by assumed bounds on the quantities in the previous step and the bounds established in the derivation of the bound on m'_n above.

Step 5. *Finally, we verify the conditions of Assumption 3.2(c) from Chernozhukov et al. (2018). Starting with $r_n = \sup_{\eta\in\mathcal{T}_n}\|E[\psi^a(\mathbf{W};\eta)] - E[\psi^a(\mathbf{W};\eta_0)]\|$,*

$$
\begin{aligned}
\|E[\psi^a(\mathbf{W};\eta)] - E[\psi^a(\mathbf{W};\eta_0)]\| &= \|E[\psi^a(\mathbf{W};\eta) - \psi^a(\mathbf{W};\eta_0)]\| \\
&= \|E[-(\mathbf{X} - \mathbf{m}(S))(\mathbf{X} - \mathbf{m}(S))^T + \mathbf{V}\mathbf{V}^T]\| \\
&\leqslant \|E[\mathbf{V}(\mathbf{m}_0(\mathbf{S}) - \mathbf{m}(\mathbf{S}))^T]\| + \|E[(\mathbf{m}_0(\mathbf{S}) - \mathbf{m}(\mathbf{S}))\mathbf{V}^T]\| \\
&\qquad + \|E[(\mathbf{m}_0(\mathbf{S}) - \mathbf{m}(\mathbf{S}))(\mathbf{m}_0(\mathbf{S}) - \mathbf{m}(\mathbf{S}))^T]\| \\
&\leqslant 2E\big[\|\mathbf{V}\| \cdot \|(\mathbf{m}_0(\mathbf{S}) - \mathbf{m}(\mathbf{S}))\|\big] + E\big[\|(\mathbf{m}_0(\mathbf{S}) - \mathbf{m}(\mathbf{S}))\|^2\big] \\
&\leqslant 2\sqrt{E\big[\|\mathbf{V}\|^2\big]E\big[\|\mathbf{m}_0(\mathbf{S}) - \mathbf{m}(\mathbf{S})\|^2\big]} + E\big[\|\mathbf{m}_0(\mathbf{S}) - \mathbf{m}(\mathbf{S})\|^2\big] \\
&= 2\big\|\|\mathbf{V}\|\big\|_{P,2} \cdot \big\|\|\mathbf{m}_0(\mathbf{S}) - \mathbf{m}(\mathbf{S})\|\big\|_{P,2} + \big\|\mathbf{m}_0(\mathbf{S}) - \mathbf{m}(\mathbf{S})\|\big\|^2_{P,2} \\
&\leqslant (pC)(p\delta_n) + (p^2C\delta_n) \\
&\leqslant \delta'_n
\end{aligned}
$$

Where the first inequality is by the triangle inequality, the second inequality is by Jensen's inequality, the third inequality is by Cauchy-Schwarz, and the following inequality by the assumption that $\|V_j\|_{P,2} < C$ and $\|m_{0j} - m_j\|_{P,2} \leqslant \delta_n$ and $\|m_{0j} - m_j\|_{P,q} \leqslant C$ for $\eta \in \mathcal{T}_n$ for $j = 1, ..., p$ and $q > 4$ (since $\|f\|_{P,q_1} \leqslant \|f\|_{P,q_2}$ for $0 < q_1 < q_2 < \infty$ by Jensen's inequality with $\phi(x) = |x|^{q_2/q_1}$). This establishes the bound on r_n.

Next we establish the bound on $r'_n = \sup_{\eta\in\mathcal{T}_n}(E[\|\psi(\mathbf{W};\boldsymbol{\beta}_0,\eta) - \psi(\mathbf{W};\boldsymbol{\beta}_0,\eta_0)\|^2])^{1/2}$:

$$
\begin{aligned}
\left(E[\|\psi(\mathbf{W};\boldsymbol{\beta}_0,\eta)-\psi(\mathbf{W};\boldsymbol{\beta}_0,\eta_0)\|^2]\right)^{1/2} &= \big\|\|\psi(\mathbf{W};\boldsymbol{\beta}_0,\eta)-\psi(\mathbf{W};\boldsymbol{\beta}_0,\eta_0)\|\big\|_{P,2}\\
&= \big\|\|\{Y-\ell(\mathbf{S})-(\mathbf{X}-\mathbf{m}(\mathbf{S}))^T\boldsymbol{\beta}_0\}(\mathbf{X}-\mathbf{m}(\mathbf{S}))-\\
&\qquad \{Y-\ell_0(\mathbf{S})-(\mathbf{X}-\mathbf{m}_0(\mathbf{S}))^T\boldsymbol{\beta}_0\}(\mathbf{X}-\mathbf{m}_0(\mathbf{S}))\|\big\|_{P,2}\\
&\leqslant \big\|\|U(\mathbf{m}_0(\mathbf{S})-\mathbf{m}_0(\mathbf{S}))\|+\|(\ell_0(\mathbf{S})-\ell(\mathbf{S}))\mathbf{V}\|\\
&\qquad +\|(\ell_0(\mathbf{S})-\ell(\mathbf{S}))(\mathbf{m}_0(\mathbf{S})-\mathbf{m}_0(\mathbf{S}))\|\\
&\qquad +\|\mathbf{V}(\mathbf{m}_0(\mathbf{S})-\mathbf{m}(\mathbf{S}))^T\boldsymbol{\beta}_0\|\\
&\qquad +\|(\mathbf{m}_0(\mathbf{S})-\mathbf{m}(\mathbf{S}))(\mathbf{m}_0(\mathbf{S})-\mathbf{m}(\mathbf{S}))^T\boldsymbol{\beta}_0\|\big\|_{P,2}\\
&\leqslant \big\||U|\cdot\|(\mathbf{m}_0(\mathbf{S})-\mathbf{m}_0(\mathbf{S}))\|\big\|_{P,2}+\big\||\ell_0(\mathbf{S})-\ell(\mathbf{S})|\cdot\|\mathbf{V}\|\big\|_{P,2}\\
&\qquad +\big\||\ell_0(\mathbf{S})-\ell(\mathbf{S})|\cdot\|(\mathbf{m}_0(\mathbf{S})-\mathbf{m}_0(\mathbf{S}))\|\big\|_{P,2}\\
&\qquad +\big\|\|\mathbf{V}(\mathbf{m}_0(\mathbf{S})-\mathbf{m}(\mathbf{S}))^T\boldsymbol{\beta}_0\|\big\|_{P,2}\\
&\qquad +\big\|\|(\mathbf{m}_0(\mathbf{S})-\mathbf{m}(\mathbf{S}))(\mathbf{m}_0(\mathbf{S})-\mathbf{m}(\mathbf{S}))^T\boldsymbol{\beta}_0\|\big\|_{P,2}\\
&\leqslant \big\||U|\cdot\|(\mathbf{m}_0(\mathbf{S})-\mathbf{m}_0(\mathbf{S}))\|\big\|_{P,2}+\big\||\ell_0(\mathbf{S})-\ell(\mathbf{S})|\cdot\|\mathbf{V}\|\big\|_{P,2}\\
&\qquad +\big\||\ell_0(\mathbf{S})-\ell(\mathbf{S})|\cdot\|(\mathbf{m}_0(\mathbf{S})-\mathbf{m}_0(\mathbf{S}))\|\big\|_{P,2}\\
&\qquad +\big\|\|\mathbf{V}\|\cdot\|\mathbf{m}_0(\mathbf{S})-\mathbf{m}(\mathbf{S})\|\big\|_{P,2}\|\boldsymbol{\beta}_0\|\\
&\qquad +\big\|\|\mathbf{m}_0(\mathbf{S})-\mathbf{m}(\mathbf{S})\|^2\big\|_{P,2}\|\boldsymbol{\beta}_0\|\\
&\leqslant \sqrt{C}p\delta_n+\sqrt{pC}\delta_n+pC\delta_n+\sqrt{pC}p\delta_nC+\sqrt{p}Cp\delta_nC\\
&= (\sqrt{C}p+\sqrt{pC}+pC+\sqrt{pC}pC+\sqrt{p}C^2p)\delta_n\\
&\leqslant \delta_n'
\end{aligned}
$$

as desired, where the final inequality is due to the assumptions that $\|E[U^2|\mathbf{S}]\|_{P,\infty}\leqslant C$, $\|E[V_j^2|\mathbf{S}]\|_{P,\infty}\leqslant C$ *for* $j=1,...,p$, *and* $\|\eta_0-\eta\|_{P,\infty}\leqslant C$ *for* $\eta\in\mathcal{T}_n$, *and applying the law of iterated expectation.*

Finally we check the condition that $\lambda_n'=\sup_{r\in(0,1),\eta\in\mathcal{T}_n}\|\partial_r^2E[\psi(\mathbf{W};\boldsymbol{\beta}_0,\eta_0+r(\eta-\eta_0))]\|\leqslant\delta_n'/\sqrt{n}$. *Define* $f(r):=E[\psi(\mathbf{W};\boldsymbol{\beta}_0,\eta_0+r(\eta-\eta_0)],r\in(0,1)$. *Then for* $r\in(0,1)$,

$$
\begin{aligned}
\delta_r^2f(r) &= E\big[2(\ell(\mathbf{S})-\ell_0(\mathbf{S}))\times(\mathbf{m}(\mathbf{S})-\mathbf{m}_0(\mathbf{S}))-2(\mathbf{m}(\mathbf{S})-\mathbf{m}_0(\mathbf{S}))(\mathbf{m}(\mathbf{S})-\mathbf{m}_0(\mathbf{S}))^T\boldsymbol{\beta}_0\big]\\
&= 2E\big[(\ell(\mathbf{S})-\ell_0(\mathbf{S}))\times(\mathbf{m}(\mathbf{S})-\mathbf{m}_0(\mathbf{S}))\big]-2E\big[(\mathbf{m}(\mathbf{S})-\mathbf{m}_0(\mathbf{S}))(\mathbf{m}(\mathbf{S})-\mathbf{m}_0(\mathbf{S}))^T\boldsymbol{\beta}_0\big]
\end{aligned}
$$

Then note that:

$$
\begin{aligned}
\|E\big[(\ell(\mathbf{S})-\ell_0(\mathbf{S}))\times(\mathbf{m}(\mathbf{S})-\mathbf{m}_0(\mathbf{S}))\big]\| &= \sqrt{\sum_{j=1}^p E\big[(\ell(\mathbf{S})-\ell_0(\mathbf{S}))\times(m_j(\mathbf{S})-m_{0j}(\mathbf{S}))\big]^2}\\
&\leqslant \sqrt{\sum_{j=1}^p E\big[(\ell(\mathbf{S})-\ell_0(\mathbf{S}))^2\big]\times E\big[(m_j(\mathbf{S})-m_{0j}(\mathbf{S}))^2\big]}\\
&= \sqrt{\sum_{j=1}^p \|\ell-\ell_0\|_{P,2}^2\times\|m_j-m_{0j}\|_{P,2}^2}
\end{aligned}
$$

For $\eta \in T$, $\|\ell - \ell_0\|_{P,2}^2 \times \|m_j - m_{0j}\|_{P,2}^2 \leqslant \delta_n^2 n^{-1}$, so:

$$\begin{aligned}\|E\big[(\ell(\mathbf{S}) - \ell_0(\mathbf{S})) \times (\mathbf{m}(\mathbf{S}) - \mathbf{m}_0(\mathbf{S}))\big]\| &\leqslant \sqrt{\sum_{j=1}^{p} \delta_n^2 n^{-1}} \\ &= \sqrt{p\delta_n^2 n^{-1}} \\ &= \sqrt{p}\delta_n n^{-1/2}\end{aligned}$$

By the same reasoning, $\|E\big[(\mathbf{m}(\mathbf{S}) - \mathbf{m}_0(\mathbf{S}))^2\big]\| \leqslant \sqrt{p}\delta_n n^{-1/2}$. Then, we have that $\|\partial_r^2 E[\psi(\mathbf{W}; \boldsymbol{\beta}_0, \eta_0 + r(\eta - \eta_0))]\| = \|2E\big[(\ell(\mathbf{S}) - \ell_0(\mathbf{S})) \times (\mathbf{m}(\mathbf{S}) - \mathbf{m}_0(\mathbf{S}))\big] - 2E\big[(\mathbf{m}(\mathbf{S}) - \mathbf{m}_0(\mathbf{S}))(\mathbf{m}(\mathbf{S}) - \mathbf{m}_0(\mathbf{S}))^T\boldsymbol{\beta}_0\big]\| \leqslant 2\|E\big[(\ell(\mathbf{S}) - \ell_0(\mathbf{S})) \times (\mathbf{m}(\mathbf{S}) - \mathbf{m}_0(\mathbf{S}))\big]\| + 2\|E\big[(\mathbf{m}(\mathbf{S}) - \mathbf{m}_0(\mathbf{S}))(\mathbf{m}(\mathbf{S}) - \mathbf{m}_0(\mathbf{S}))^T\boldsymbol{\beta}_0\big]\|$, which does not depend on r, so that for $\eta \in T$, $\|\partial_r^2 E[\psi(\mathbf{W}; \boldsymbol{\beta}_0, \eta_0 + r(\eta - \eta_0))]\| \leqslant 4\sqrt{p}\delta_n n^{-1/2} \leqslant \delta_n' n^{-1/2}$, establishing the desired bound on λ_n'.

With the conditions of Assumptions 3.1 and 3.2 from Chernozhukov et al. (2018) verified, Lemma 1 follows from Facts 3.1 and 3.2 from Chernozhukov et al. (2018).

S6.2 Double Spatial Regression model

Lemma 2 follows directly from Theorem 3.3 from (Eberts and Steinwart, 2013), using $\rho = \ln(n)$. Note that the estimated function from the least-squares SVM with Gaussian kernel and least-squares loss analyzed in (Eberts and Steinwart, 2013) is identical to the posterior mean of a Gaussian process with Gaussian kernel; see e.g. (Kanagawa et al., 2018).

Lemma 2 (Convergence rate for bounded regression using GP posterior mean) *Let $\widehat{f}$ be an estimate of $f(S) = E(Y|S)$ obtained by a Gaussian process posterior mean, with Gaussian kernel, using parameters γ_n, λ_n selected by the training-validation scheme in (Eberts and Steinwart, 2013) using grids as specified in Algorithm 1. Let P_S be the marginal distribution of S over $\mathbb{R}^d$ with support in the $\|\cdot\|_2$-unit ball. Let the density of P_S be $p_S \in L_q(\mathbb{R}^d)$ for some $q \geqslant 1$, and let $f \in L_2(\mathbb{R}^d) \cap L_\infty(\mathbb{R}^d)$ and $f \in B_{2s,\infty}^\alpha$ for $\alpha \geqslant 1$ and $s \geqslant 1$ such that $\frac{1}{q} + \frac{1}{s} = 1$. Let $Y \in [-M, M], M > 0$ and let $\widehat{f}$ be clipped at $-M, M$.*

Then with probability no less than $1 - \frac{1}{n}$, $\|\widehat{f} - f\|_{P,2} \leqslant C\log(n)n^{-\frac{\alpha}{2\alpha+d}+\xi}$, for all $\xi > 0$ and some $C > 0$.

Lemma 3 follows directly from Theorem 3.6 from (Eberts and Steinwart, 2013), using $\widehat{\rho} = \ln(n)$ and $\overline{\rho} = \ln(n)$ and clipping the absolute value of the fitted function at $\min\{1, M_n\}$ rather than simply M_n, which does not change the result since by assumption the true function lies in $[-1, 1]$. Note that per the proof of Theorem 3.6, the constant C in the original statement of Theorem 3.6 does not depend on either $\widehat{\rho}$ or $\overline{\rho}$ allowing these substitutions.

Lemma 3 (Convergence rate for regression with normal errors using GP posterior mean) *Let $\widehat{f}$ be an estimate of $f(S) = E(Y|S)$ obtained by a Gaussian process posterior mean, with Gaussian kernel, using parameters γ_n, λ_n selected by the training-validation scheme in (Eberts and Steinwart, 2013) using grids as specified in Algorithm 1. Let P_S be the marginal distribution of S over $\mathbb{R}^d$ with support in the $\|\cdot\|_2$-unit ball. Let the density of P_S be $p_S \in L_q(\mathbb{R}^d)$ for some $q \geqslant 1$, and let $f \in L_2(\mathbb{R}^d) \cap L_\infty(\mathbb{R}^d)$ and $f \in B_{2s,\infty}^\alpha$ for $\alpha \geqslant 1$ and $s \geqslant 1$ such that $\frac{1}{q} + \frac{1}{s} = 1$. Assume further that $f(S) \in [-1, 1]$.*

Let $Y_i = f(S_i) + \epsilon_i$ where $\epsilon_i \sim$ ind. $N(0, \sigma_i^2)$, and let there exist some constant C_0 such that all $\sigma_i^2 < C_0$. Let $\widehat{f}$ be clipped so that $|\widehat{f}| \leqslant \min\{1, M_n\}$ where $M_n = 4\sqrt{C_0}\sqrt{\ln(n)}$.

Then with probability no less than $1 - \frac{2}{n}$, $\|\widehat{f} - f\|_{P,2} \leqslant C\log(n)n^{-\frac{\alpha}{2\alpha+2}+\xi}$, for all $\xi > 0$ and some $C > 0$.

The proof of Theorem 1 follows by verifying Assumption 1 using assumptions A1-A6 and Lemmas 1-3.

Proof 2 (Proof of Theorem 1) *First note that estimation of $\boldsymbol{\beta}_0$ by $\widehat{\boldsymbol{\beta}}_{DSR}$ using Algorithm 1 is equivalent to using DML2 in Definition 3.2 of Chernozhukov et al. (2018), under the Partially Linear Regression DML*

estimation established by Lemma 1, and estimation of the nuisance parameters uses the same Gaussian Process (GP) estimates used in Lemmas 2 and 3. Therefore, the result of Theorem 1 follows from satisfying Assumption 1, which in turn is achieved in part by satisfying the assumptions for Lemmas 2 and 3 which establish the necessary convergence rates for prediction using GP regression.

In Assumption 1, (a) follows by Assumption A1, (b) follows from the assumption of bounded or normal distributions of U, V_j, so that all moments are finite, and the assumption that $\boldsymbol{\beta}_0 \in \mathbb{R}^p$, and (c) and (d) follow from Assumption A2.

To satisfy (e) in Assumption 1, first note that by Assumption A4 the components of η_0 are bounded in some interval, and that the nuisance parameter estimates are clipped accordingly to reside in some interval, satisfying $\|\eta_0 - \widehat{\eta}_0\|_{P,\infty} < C$ for some $C > 0$ (recall the notation $\|\eta_0 - \widehat{\eta}_0\|_{P,q} = \max_j \|\eta_{0j} - \widehat{\eta}_{0j}\|_{P,q}$ for $q \in [0, \infty)$). Next apply Lemmas 2 and 3 to satisfy the convergence rate requirements. Let $\alpha_X \geqslant \frac{d}{2}$ and $\alpha_X > 1$ per Assumption A6. Then if each m_{j0} is estimated by $\widehat{m}_{j0}$ using a Gaussian process mean with clipping at appropriate bounds as in Lemmas 2 and 3, then with probability no less than $1 - \frac{2}{n}$, $\|\widehat{m}_{j0} - m_{j0}\|_{P,2} \leqslant C_j \log(n) n^{-\frac{\alpha_X}{2\alpha_X + d} + \xi}$ for any $\xi > 0$ and some $C_j > 0$, and $n^{-\frac{\alpha_X}{2\alpha_X+d}} < n^{-1/4}$. Let $\gamma = \frac{\alpha_X}{2\alpha_X + d} - 1/4 > 0$. Then with probability no less than $1 - \frac{2}{n}$, $\|\widehat{m}_{j0} - m_{j0}\|_{P,2} \leqslant C_j \log(n) n^{-1/4} n^{-\gamma} n^{\xi}$, for all $\xi > 0$. Pick $\xi < \gamma$. Let $\gamma^* = \gamma - \xi > 0$. This holds for $j = 1, ..., p$; let C_m be greater than or equal to all C_j. Then letting $\delta'_n = C_m(\log(n) n^{-\gamma^*}) \vee n^{-1/4} \to 0$, we have that $\|\widehat{m}_{j0} - m_{j0}\|_{P,2} \leqslant \delta'_n n^{-1/4}$ with probability no less than $1 - \frac{2}{n}$ for all m_{0j}, $j = 1, ..., p$, and if $\alpha_Y \geqslant \frac{d}{2}$ and $\alpha_Y > \frac{d^2}{4\alpha_X}$, the analogous result holds for g_0 as well.

Since all of the errors (of estimates of $g_0, m_{01}, ..., m_{0p}$) individually obey the desired rates, each with marginal probability no less than $1 - \frac{2}{n}$, there exists a sequence $\Delta \to 0$ such that with probability no less than $1 - \Delta_n$, all estimates of $g_0, m_{01}, ..., m_{0p}$ simultaneously obey the desired error bounds. To see why, let A_{kn}, $k = 1, ..., K$, $n = 1, 2, ...$ be a finite number K of sequences of events, such that $P(A_{kn}) \to 1$ as $n \to \infty$ for each k. $P(A_{1n} \cup A_{2n}) = P(A_{1n}) + P(A_{2n}) - P(A_{1n} \cap A_{2n})$, and since $P(A_{1n} \cup A_{2n}) \geqslant P(A_{1n}), P(A_{2n})$, we have that $P(A_{1n} \cup A_{2n}) \to 1$ as $n \to \infty$. Hence, $\lim_{n\to\infty} P(A_{1n} \cap A_{2n}) = \lim_{n\to\infty} P(A_{1n}) + \lim_{n\to\infty} P(A_{2n}) - \lim_{n\to\infty} P(A_{1n} \cup A_{2n}) = 1$. Applying induction establishes that $\lim_{n\to\infty} P(A_{1n} \cap A_{2n} \cap ... \cap A_{Kn}) = 1$. Therefore, there exists some sequence $L_n \to 0$ such that $P(A_{1n} \cap A_{2n} \cap ... \cap A_{Kn}) \geqslant 1 - L_n$.

Therefore, there exists some sequence $\Delta_n \to 0$ such that $\|\widehat{g}_0 - g_0\|_{P,2} \times \|\widehat{m}_{0j} - m_{0j}\|_{P,2} \leqslant C\delta_n n^{-\frac{1}{2}}$, $\|\widehat{m}_{0j} - m_{0j}\|^2_{P,2} \leqslant C\delta_n n^{-\frac{1}{2}}$, and $\|\widehat{\eta}_0 - \eta_0\|_{P,2} \leqslant \delta_n$ for $j = 1, ..., p$, with $\delta_n = \delta'^2_n \geqslant n^{-1/2}$, with probability no less than $1 - \Delta_n$.

Thus part (e) of Assumption 1 is satisfied.

With parts (a)-(e) of Assumption 1 satisfied, apply Lemma 1 to obtain $\widehat{Var}(\widehat{\boldsymbol{\beta}}_0)^{-1/2}(\widehat{\boldsymbol{\beta}}_0 - \boldsymbol{\beta}_0) \xrightarrow{D} N(\mathbf{0}, \mathbf{I}_p)$.

References

Andrews, D. W. K. (1994). Asymptotics for semiparametric econometric models via stochastic equicontinuity. *Econometrica* **62,** 43–72.

Belloni, A., Chernozhukov, V., Fernández-Val, I., and Hansen, C. (2017). Program evaluation and causal inference with high-dimensional data. *Econometrica* **85,** 233–298.

Chernozhukov, V., Chetverikov, D., Demirer, M., Duflo, E., Hansen, C., Newey, W., and Robins, J. (2018). Double/debiased machine learning for treatment and structural parameters. *The Econometrics Journal* **21,** C1–C68.

Chipman, H. A., George, E. I., and McCulloch, R. E. (2010). BART: Bayesian additive regression trees. *The Annals of Applied Statistics* **4,** 266 – 298.

Dorie, V. (2024). *dbarts: Discrete Bayesian Additive Regression Trees Sampler.* R package version 0.9-26.

Dupont, E., Wood, S. N., and Augustin, N. H. (2022). Spatial+: A novel approach to spatial confounding. *Biometrics* **78,** 1279–1290.

Eberts, M. and Steinwart, I. (2013). Optimal regression rates for SVMs using Gaussian kernels. *Electronic Journal of Statistics* **7,** 1 – 42.

Gilbert, B., Datta, A., Casey, J. A., and Ogburn, E. L. (2021). A causal inference framework for spatial confounding. *arXiv preprint arXiv:2112.14946* .

Guan, Y., Page, G. L., Reich, B. J., Ventrucci, M., and Yang, S. (2022). Spectral adjustment for spatial confounding. *Biometrika* **110,** 699–719.

Guinness, J. (2018). Permutation and grouping methods for sharpening Gaussian Process approximations. *Technometrics* **60,** 415–429. PMID: 31447491.

Huiying Mao, R. M. and Reich, B. J. (2023). Valid model-free spatial prediction. *Journal of the American Statistical Association* **0,** 1–11.

Kanagawa, M., Hennig, P., Sejdinovic, D., and Sriperumbudur, B. K. (2018). Gaussian processes and kernel methods: A review on connections and equivalences. *arXiv preprint arXiv:1807.02582* .

Paciorek, C. J. (2010). The importance of scale for spatial-confounding bias and precision of spatial regression estimators. *Statistical Science* **25,** 107–125.

Rasmussen, C. E. and Williams, C. K. I. (2005). *Gaussian Processes for Machine Learning*. The MIT Press.

Reich, B. J., Hodges, J. S., and Zadnik, V. (2006). Effects of residual smoothing on the posterior of the fixed effects in disease-mapping models. *Biometrics* **62,** 1197–1206.

Robinson, P. M. (1988). Root-n-consistent semiparametric regression. *Econometrica* **56,** 931–954.

Stein, M. (1999). *Interpolation of Spatial Data: Some Theory for Kriging*. Springer Series in Statistics. Springer New York.

Thaden, H. and Kneib, T. (2018). Structural equation models for dealing with spatial confounding. *The American Statistician* **72,** 239–252.

Wood, S. N. (2011). Fast stable restricted maximum likelihood and marginal likelihood estimation of semiparametric generalized linear models. *Journal of the Royal Statistical Society: Series B (Statistical Methodology)* **73,** 3–36.

Wood, S. N. (2017). *Generalized Additive Models: An Introduction With R, Second Edition*. CRC press.
